# Social determinants of health and knowledge, attitude and practice studies on antimicrobial resistance—an evidence review and future direction

**DOI:** 10.1093/jacamr/dlaf141

**Published:** 2025-09-04

**Authors:** Alison Shutt, Arunima Sehgal Mukherjee, Vrinda Nampoothiri, Alison Holmes, Esmita Charani

**Affiliations:** National Institute for Health Research, Health Protection Research Unit in Healthcare Associated Infection and Antimicrobial Resistance, Imperial College London, London, UK; Centre for Sustainable Healthcare, University of Oslo, Oslo, Norway; Department of Infection Control and Epidemiology, Amrita Institute of Medical Sciences, Faridabad, Haryana, India; National Institute for Health Research, Health Protection Research Unit in Healthcare Associated Infection and Antimicrobial Resistance, Imperial College London, London, UK; Faculty of Life and Health Sciences, University of Liverpool, Liverpool, UK; The Fleming Initiative, Imperial College London and Imperial College Healthcare NHS Trust, London, UK; Faculty of Life and Health Sciences, University of Liverpool, Liverpool, UK; Division of Infectious Diseases & HIV Medicine, Department of Medicine, Groote Schuur Hospital, University of Cape Town, Cape Town, South Africa

## Abstract

**Background:**

Antimicrobial resistance (AMR) intersects with social determinants of health (SDoH) influencing individual and population infectious disease exposure, spread and outcomes. A decade after the WHO recommended global AMR awareness campaigns to assess people’s knowledge, attitude and practices (KAP), we reviewed existing KAP studies conducted among populations considered vulnerable, marginalized or deprived by SDoH indicators.

**Methods:**

We conducted a sub-analysis of KAP studies from a broader systematic review that explored the intersection of SDoH, AMR, infections and vulnerable populations. We searched Ovid MEDLINE, Ovid Embase, the Cochrane Library, PsycINFO and Scopus for studies published in English between 2000 and 2022. Titles, abstracts and full texts were screened, and qualitative analysis performed using thematic coding. Key findings were synthesized using a Strengths, Weaknesses, Opportunities and Threats (SWOT) framework.

**Results:**

Of the 126 papers included in the original review, 40 KAP studies met the inclusion criteria for this sub-analysis. Studies reported limited public knowledge about AMR and widespread practices of antibiotic self-medication. Existing recommendations emphasized continued health education. Although KAP studies describe ‘what’ antibiotic use behaviours are prevalent, they rarely address ‘why’ these behaviours occur—particularly in the context of SDoH and cultural norms. There is a pressing need to shift the research focus toward upstream drivers of behaviour, rather than solely on individual actions.

**Conclusions:**

Current KAP studies prioritize individual behaviours without adequately considering the broader social, structural and environmental determinants. While these upstream factors remain unaddressed, the practice of sub-optimal self-medication is likely to persist, despite ongoing education efforts.

## Introduction

Antimicrobial resistance (AMR) is a complex global public health challenge with considerable impact on people and the economy.^1–3^ Although globally AMR can affect anyone, people affected by poverty and health inequalities are at an increased risk.^4^ The distribution of AMR is uneven, with people in low-income countries (LICs) and lower-middle-income countries (LMICs) being more adversely affected due to inadequate access to clean water, sanitation and hygiene (WASH) and universal healthcare.^5–7^ This inequality is emphasized with 88% of deaths attributable to resistant bacterial infections located in LMICs.^2,6^ Social determinants of health (SDoH) include education, employment, housing, living environment and an individual’s age, gender, race and ethnicity.^8,9^ These determinants impact both population and individual levels of infectious disease exposure, including risks to AMR pathogens leading to poor outcomes.^6,8,9^ Recognition of the public health risk of AMR varies amongst different populations and is linked to literacy.^10,11^

In 2015, the WHO developed a Global Action Plan (GAP) for AMR.^12^ Objective One of the GAP stated: ‘Improve awareness and understanding of antimicrobial resistance through effective communication, education and training.’^12^ Recommendations included public awareness campaigns, annual World Antibiotic Awareness campaigns, facilitating behaviour change for antibiotic stewardship, and incorporating training about AMR into the curricula for schoolchildren and students. The WHO GAP was the precursor for country-specific National Action Plans (NAPs), which included public campaigns, education and recommendations for knowledge, attitude and practice (KAP) studies.^13^ To date, not all countries have a NAP and, in some countries progress has been slow and has been hampered by inadequate funding and stakeholder engagement.^13^

Despite WHO endorsement, concerns have been raised regarding the effectiveness of KAP studies.^14,15^ Questions persist about whether educational programmes result in long-term behaviour change.^16^ Although KAP studies are often used as accessible tools to assess health behaviours,^17^ they may oversimplify the complex intersecting factors—such as access, literacy and socio-environmental conditions—that shape antibiotic use.^18^

In this study, we conducted a secondary analysis of data from a systematic review^19^ to (i) identify the range and scope of KAP studies on AMR involving populations defined as vulnerable, marginalized or deprived by SDoH indicators, and (ii) examine how key findings from these studies relate to SDoH and antibiotic use. We defined vulnerable populations as individuals from low socioeconomic backgrounds, ethnic minority groups, the homeless, refugees, asylum seekers, recently arrived migrants, displaced people and the disabled. Through this work we provide recommendations to address the identified gaps and opportunities, specifically focused on vulnerable, deprived and marginalized populations.

## Methods

**Figure 1.**
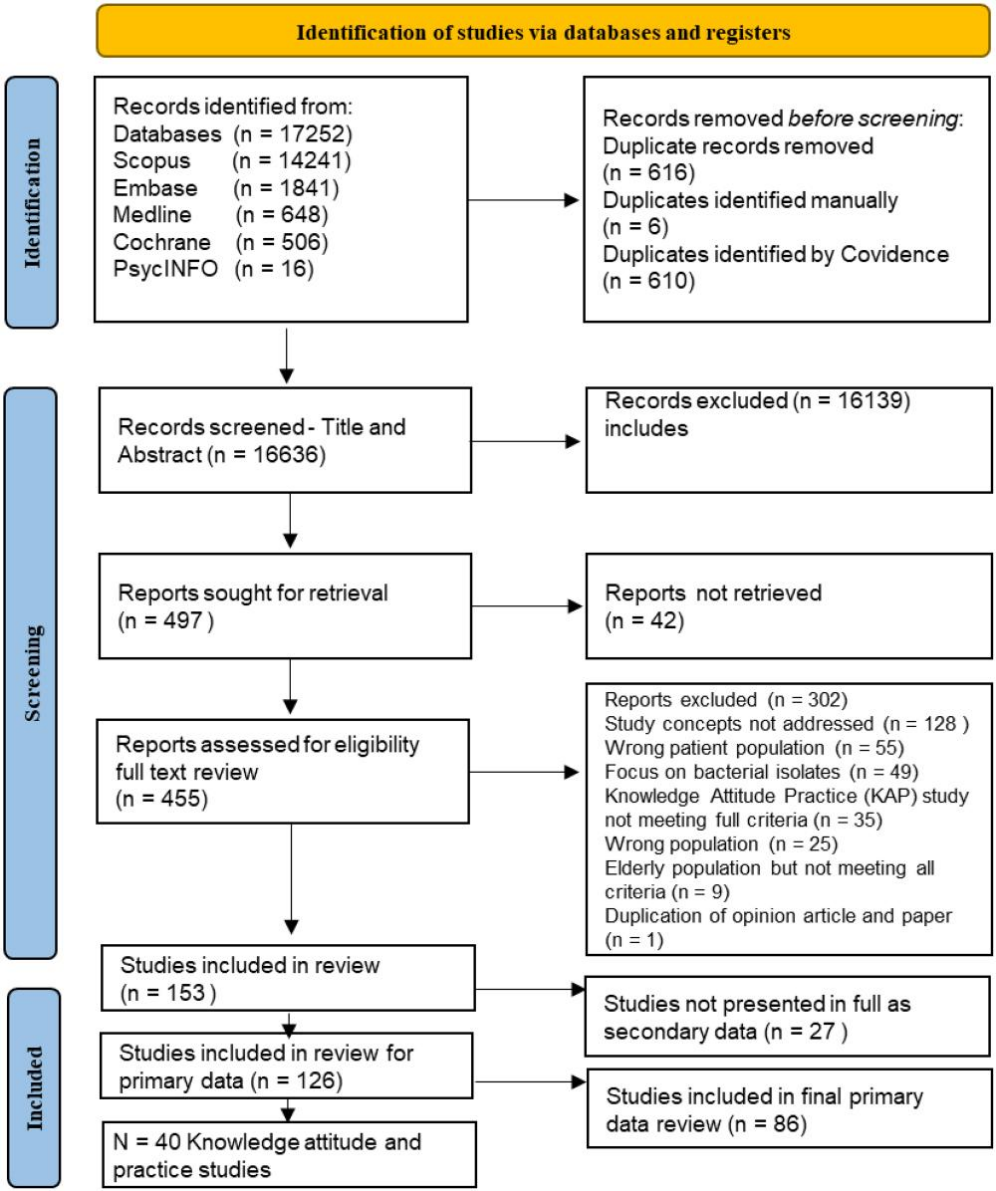
PRISMA flow diagram for the studies that included specific component parts of the KAP framework.^19^

This study used a subset of the studies extracted as part of a systematic review that examined the available evidence on the SDoH and health-seeking behaviours of vulnerable populations at risk of AMR.^19^ The systematic review protocol was registered with PROSPERO and provides the inclusion and exclusion criteria.^20^ The PRISMA methodology was followed, and searches were undertaken using Ovid MEDLINE, Ovid Embase, the Cochrane Library, PsycINFO and Scopus for published material in English, between 2000 and 2022. The search string is provided in Appendix S1 (available as Supplementary data at  *JACAMR*  Online). Title, abstract and full-text review were undertaken by a group of assessors using Covidence software. As outlined in Figure 1, the 126 publications that were included in the systematic review related to four domains, including (i) the SDoH, (ii) health-seeking behaviour, (iii) populations considered vulnerable/deprived/marginalized, and (iv) AMR and defined infections.^19^ If a study included a combination of the stated population as well as healthcare workers it was included if it met all the other stated inclusion criteria. If the study only looked at healthcare workers, the study was excluded.^19^ Studies that included specific component parts of the KAP framework were identified.

Several theoretical lenses were used to develop a composite framework for analysis of the included studies. The SDoH model as defined by Dahlgren and Whitehead^21^ was adopted to examine (i) the impact of SDoH at a policy level and individual level, and (ii) the relationship between education and a person’s knowledge. The latter point is complex as a person’s knowledge affects their beliefs and attitudes, which further impact their behaviour. To better understand these complexities and the potential causal links between attitudes, beliefs and behaviour, the Health Belief Model^22^ and the Fishbein Ajzen Behavioural Intentions Model^23^ were used. Maslow’s Hierarchy of Needs^24^ and Amartya Sen’s Capability Approach^25^ were adopted to evaluate the potential effect of SDoH, inequalities and agency on behaviour.

NVivo (version 14) software was used to qualitatively analyse the results using thematic coding. Deductive and inductive analysis was undertaken to develop the coding framework.^26^ Following analysis of the extracted data, the findings were evaluated using a Strengths, Weaknesses, Opportunities, and Threats (SWOT) framework. Recommendations were developed from this framework.

## Results

**Figure 2.**
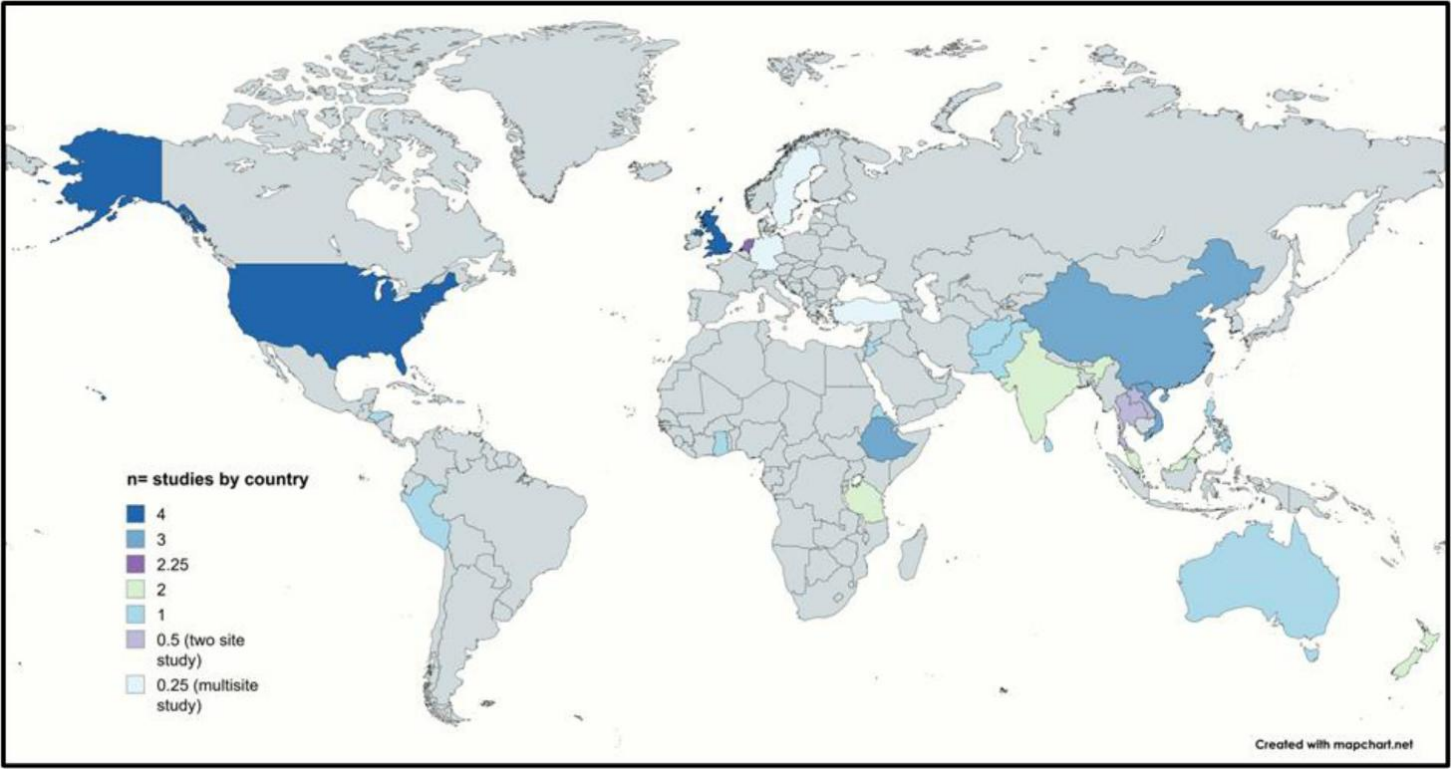
Distribution of the KAP studies in vulnerable populations as defined by SDoH indicators, by country.

Forty studies were identified. The research methods in the included studies were 24/40 (60%) quantitative, 12/40 (30%) qualitative, and 4/40 (10%) mixed methods. The number of studies by country are outlined in Figure 2. The distribution of studies by income category were high-income countries (HICs) 15/40 (37.5%), LMICs 12.5/40 (31.25%), upper-middle-income countries (UMICs) 7.5/40 (18.75%) and LICs 5/40 (12.5%). The studies by individual country include the USA 4/40, UK 4/40, Ethiopia 3/40, China 3/40, Vietnam 3/40, Netherlands 2.25/40, Malaysia 2/,40, Tanzania 2/40, New Zealand 2/40, India 2/40, Sri Lanka 1/40, Peru 1/40, Australia 1/40, Eritrea 1/40, Philippines 1/40, Afghanistan 1/40, Honduras 1/40, Ghana 1/40, Puerto Rico 1/40, Pakistan 1/40, Jordan 1/40, Thailand 0.5/40, Laos 0.5/40, Germany 0.25/40, Turkey 0.25/40 and Sweden 0.25/40. A study with less than one is a multi-centre study. The distribution of studies by WHO region^27^ include the African region 4/40, Americas region 4/40, South-East Asia region 4/40, European region 5/40, Eastern Mediterranean region 3/40 and Western Pacific region 12/40.

Table 1 outlines the main findings from the included studies. The overarching findings by KAP category are provided in the sections below.

**Table 1.** Summary of the KAP studies that include populations defined as vulnerable, deprived and marginalized by social determinants of health indicators

Author, year, country/region	Population sample	Population demographics and social determinants of health	Evidence of health-seeking behaviour	Infection-related data	Study design and aim	Key findings
Afari-Asiedu S *et al.* 2020^28^ Ghana, Kintampo North and South Districts	55 Ghanaian men and women community members from rural forest and savannah areas	Age, sex, occupation, knowledge, cultural practices including rituals	Obtaining medication from hospitals, pharmacies and unlicensed vendors	Proxy: use and obtaining antibiotics	Qualitative, exploratory, ethnomethodology study including focus groups and in-depth interviews to explore inappropriate antibiotic use and confusing antibiotics with other medicines	Lack of knowledge about what antibiotics are and their respective use. No appropriate terminology in the local dialect. Participants described antibiotics by the colour codes of the capsules and perceived as painkillers. Antibiotics used for stomach pain, diarrhoea, gonorrhoea, wounds/sores, boils and headache. Knowledge obtained via previous medicines prescribed in hospitals or dispensed by pharmacies and licensed practitioners. Subsequently self-medication with the same antibiotics was practised when similar symptoms were experienced. Family members, colleagues and friends influenced self-medication. Learning from radio, television adverts and drug peddlers who visit houses to sell antibiotics
Al Baz M *et al.* 2018^29^ Jordan, Irbid, Baqaa South, Taybeh and Marka	245 Palestinian refugees (men and women) in urban and rural locations	Sex, age, education status, employment status, income level, location of residence	Attending United Nations Relief and Works Agency health centres	Proxy: antibiotic use	Quantitative, cross-sectional study to assess knowledge, attitude and behaviour regarding antibiotic use	Questions on antibiotic use, resistance and side-effects revealed a poor understanding of these factors. 34% did not know which infectious agent was targeted by antibiotics, and 20% falsely stated that antibiotics generally treat viral infections. Antibiotic usage within the study population was very high. 47% of the respondents had used antibiotics once or more in the previous 3 months. More than 25% used antibiotics four times or more in the same period. Sharing antibiotics at home (63%), over-the-counter purchases (without prescription) (60%) and using leftover antibiotics (38%) were the most prevalent forms of self-medication
Alkirawan R *et al.* 2022^30^ Netherlands national study	12 newly arrived Syrian refugees. Men *n* = 5 and women *n* = 7	Sex, age 30 to 46, level of education, former profession and health beliefs	Adapting to new health system—Dutch primary care	Antibiotic use as a proxy	Qualitative study using semi-structured interviews to explore the perspectives and expectations about antibiotic use and prescribing	In the home country obtained antibiotics on a just-in-case basis for minor illnesses and young children. Health beliefs included the curative power of antibiotics and the importance of antibiotics for recovery. Participants were dissatisfied with the Dutch healthcare system as they couldn't access medication as previously undertaken. In the Netherlands paracetamol was prescribed postoperatively rather than antibiotics as in Syria. This caused the participants to feel their condition was incorrectly assessed. Participants who had previous experience of taking antibiotics were used to repeating the antibiotics for the same symptoms without re-consulting the doctor
Barker AK *et al.* 2017^31^ India, Haryana, Sikandarpur Badha, Bhirawati, Silani, Kadipur and Pratap Nagar	20 Indian men and women from villages and roadside stalls in both rural and urban areas	Sex, age, education status, illiteracy, monthly household income, socioeconomic deprivation driving health behaviour	The impact of healthcare access, knowledge and income on antibiotic use and practices	Proxy: use of antibiotics	Qualitative study using semi-structured interviews and questionnaires to assess health literacy, antibiotic use and knowledge	Purchasing antibiotics without a prescription is illegal and only qualified doctors are allowed to prescribe antibiotics in India. However, 85% of participants reported limited access to see a doctor. Consulting a doctor in another village was not feasible due to additional costs associated with travel. Fifteen participants with income levels less than $375 went directly to the pharmacy. 65% of participants stopped taking antibiotics due to associated costs and low income. 80% of participants had children and care was prioritized for their children. Evidence of minimal understanding about antibiotics and unaware of adverse effects
Bernadas JM 2019^32^ Philippines, Manila	28 Filipino women (mothers or guardians and female senior citizens) in urban areas	Socioeconomic deprivation	Sourcing antibiotics	Proxy: obtaining and meanings of antibiotics	Qualitative study using focus groups to explore the meanings, experiences and information sources for antibiotics	Antibiotics had multiple meanings for acquisition, consumption and side-effects. Participants repeatedly stated that antibiotics were for hard-to-cure or severe diseases. Antibiotics were associated with prescription policy, i.e. obtained through prescription from doctors. Medical doctors were the most accessed and preferred sources of antibiotic-related information due to perceived trust and expertise. Experiences with antibiotics were linked with financial difficulties. All women stated that adherence to the prescription was linked with affordability of antibiotics and their respective workload. Differences in brands were linked with effectiveness and varying costs
Bogale AA *et al.* 2019^33^ Ethiopia, Addis Ababa	595 Ethiopian men and women from community urban homes	Sex, age, ethnicity, location of home, educational status, occupation and household income	Use and knowledge of antibiotics	Proxy: antibiotics	Mixed methods, cross-sectional study to assess the knowledge, attitude and practice of antibiotic self-medication	Participants—58.7% female, 8.4% illiterate, 21% monthly income less than 500ETB. 67.3% had self-medicated, determinants were age, educational status and income. 82.3% purchased antibiotics (amoxicillin and co-trimoxazole) without a prescription from a pharmacy/drug store. History of discontinuation, forgetting doses, duration, frequency and sharing antibiotics. 56.3% reported leftover antibiotics kept for future use. 60.3% outlined a previous experience as reason to use antibiotics. Misconception and misbeliefs included: 46% believed antibiotics and anti-inflammatory drugs were the same, 49.9% believed antibiotics should be used for the common cold, 47% agreed treatment would not be effective if antibiotics reduced
Burtscher D *et al.* 2021^34^ Afghanistan, Kabul	Three groups including *n* = 21 men and women over 18 years and caregivers of children, based in urban areas attending outpatients in the Ahmad Shah Baba (ASB) District Hospital, Pashto ethnicity and illiterate. Healthcare staff from the same institution (*n* = 18) and *n* = 351 patients and caretakers completed a questionnaire	Age, sex, ethnicity, education status, number of children, household size, housing location	Attending secondary care	Proxy: perceptions and use of antibiotics	Mixed methods study conducted in two stages to explore the perceptions and attitudes toward antibiotics among patients, prescribers and pharmacists	Poor knowledge of antibiotics and antibiotic resistance were drivers for inappropriate use. Perceptions—living in dirty, dusty, polluted environments increased the perceived need across all participants. Associated beliefs included pollution causing disease in the body, which required antibiotics to clean dirt from the body and blood to strengthen it. Antibiotics which had been previously used; e.g. amoxicillin was described as strong and effective and could cure disease quickly. Administration—23% stopped taking the full course as they felt better or thought they were cured. Leftover medication was either kept for future use or disposed of. Participants used leftover medication for the same condition. Unprescribed medication and advice obtained via pharmacies
Cheng J *et al.* 2018^35^ China, Anhui	2760 men and women living in rural village households	Sex, age, socioeconomic deprivation, education status, health insurance, household size	Behaviours associated with antibiotic use	Proxy: antibiotic knowledge and associated behaviours	Quantitative cross-sectional study to explore: (i) knowledge and behaviours of participants; (ii) participants’ knowledge of antibiotics distributed by sociodemographic characteristics; and (iii) the relationship between level of knowledge about antibiotics and antibiotic use behaviours	2390 (91.6%) participants believed that antibiotics could kill or control viruses. 391 (62.7%) had bought over-the-counter antibiotics. 329 (14.8%) used leftover medication from a previous illness or from a relative/friend. 1163 (44.7%) would stop taking antibiotics on relief of symptoms. 1052 (46.3%) with symptoms of common cold or gastrointestinal infection or urinary tract infection (UTIs) used leftover or over-the-counter medication. 755 (29%) were satisfied that doctors always gave a prescription. 1322 (71.5%) said they would adhere to the doctor’s decision not to prescribe antibiotics, 526 (28.5%) stated they may be unhappy and see another doctor or purchase antibiotics. Increased education and a younger age were associated with a higher knowledge score
Corbett KK *et al*. 2005^36^ USA, Colorado	992 (total): non-Hispanic Whites (692) and Hispanic Latino (300). Men and women living in urban households	Age, sex, education level, income level, internet access, children under 5 years and office visit	Proxy antibiotic knowledge and antibiotic use. Access to healthcare measured including preferred language to speak to health provider and for written material	Respiratory tract infection—upper respiratory tract infection	Quantitative cross-sectional study including a telephone survey in English or Spanish. Multivariable logistic regression analyses were conducted. The study examined relationships with ethnicity, primary language use, and antibiotic-related knowledge, attitudes and awareness	Knowledge of appropriate antibiotic use for colds and bronchitis was low. Hispanics surveyed in Spanish, compared with non-Hispanic Whites, had significantly lower knowledge about antibiotics for colds, higher knowledge for bronchitis, lower awareness about antibiotic resistance, and greater dissatisfaction if antibiotics were not prescribed. Independent predictors of awareness were ethnicity, education and age. Independent predictors of dissatisfaction were ethnicity, knowledge about antibiotic use for colds and bronchitis. Ethnicity was an independent predictor of knowledge about the inappropriateness of antibiotics for colds and bronchitis
Crigger NJ *et al.* 2004^37^ Honduras, Tegucigalpa, La Libertad	939 Honduran men and women in two rural areas including one poor and one farming community and two urban areas	Sex, age, rural and urban location, education status, literacy levels. Questions to assess poverty included owning a car, domestic help and having a living room that wasn’t used as a bedroom	Proxy obtaining and reasons for taking antibiotics. One set of data collected from rural clinic	Proxy: use of antibiotics for multiple reasons	A quantitative study using a model to assess choices and acquisition of antibiotics. Preparation to develop an educational programme for the designated population	Participants from rural areas reported a significantly lower use of antibiotics than in urban areas. Antibiotics were taken for upper respiratory symptoms, with some people taking antibiotics for diarrhoea and other gastrointestinal symptoms. Socioeconomic indicators did not show significant relationships with frequency, length or attitudes about antibiotics. Most participants consulted healthcare practitioners and obtained antibiotics from public healthcare facilities and private pharmacies
Dunn-Navarra AM *et al.* 2012^11^ USA, northern Manhattan	154 urban Latino immigrant parents	Age, sex, ethnicity, race, country of birth, time in the USA, healthcare coverage, socioeconomic deprivation, income levels, education status. Children attending head start programme, weekly hours spent away from home by parent. Health literacy abilities: Short Test of Functional Health Literacy in Adults (S-TOFHLA), and Newest Vital Sign (NVS), English proficiency. Self-reported health assessment	Access to healthcare, preferred language for healthcare	Upper respiratory tract infection	Quantitative study including a knowledge and attitude survey. The aims were to: (i) describe the influence of health literacy on parental knowledge and attitudes/beliefs for upper respiratory tract infections; and (ii) examine the correlation between two health literacy tools	Participants were predominantly female (98.7%) and Latino (91.6%) with a median age of 29 years (range 19–53 years). Participants were primarily born outside the USA (89.6%) but had resided in the USA for 5 years or more (65.6%). One in three had inadequate health literacy levels. Lower mean antibiotic knowledge scores were observed in participants with inadequate health literacy levels on both the NVS and S- TOFHLA literacy scales compared with mean scores of participants with adequate health literacy. There was lack of healthcare coverage (68.8%). Awareness and educational interventions required to increase health literacy for the stated infection
Emgard M *et al.* 2022^38^ Tanzania, Moshi Municipal, Moshi District; Kilimanjaro, Northern Tanzania	54 mothers of children under 5 years old. Dispensaries and health facilities in urban and rural areas	Socioeconomic deprivation, education level, marital status	Access to healthcare for children	Proxy: use of antibiotics	Qualitative study was undertaken with a phenomenographic approach. The aim was to understand the mothers’ perceptions of antibiotic use in their children	Three themes: (i) Antibiotics—perceived as a universal treatment for common symptoms or diseases with limited side-effects. (ii) Accessing treatment—preference to attend a healthcare facility providing free healthcare for under 5 s. However, unforeseen costs, transportation, long waits and lack of financial support from their husbands were barriers. Pharmacies were seen as cheap and convenient to access previously used or prescribed antibiotics and advice was also sought from neighbours. (iii) Administering antibiotics—mothers described behaviour that could lead to disease, e.g. playing in dirty water or inadequate hygiene
Francois Watkins LK *et al.* 2015^39^ USA national study	Survey data for Hispanic men and women. Year 2012: *n* = 4044 Year 2013: *n* = 3502. The participants represented (i) adult consumers (all ethnicities), (ii) adult Hispanic participants, and (iii) primary, secondary and tertiary healthcare providers	Sex, age, race, ethnicity, household income, household size, education, region	Proxy: antibiotic knowledge and attitudes	Upper respiratory tract infection	Quantitative study using online national survey data. The aim was to understand healthcare provider and consumer knowledge and attitudes that influence antibiotic use	Responses from Hispanic participants were different from the overall survey population. Hispanics with a cold believed antibiotics prevent more serious illness (40% vs 17%) and assist in a faster recovery (48% vs 25%). Hispanic participants were more likely to obtain antibiotics from other sources rather than from a doctor/clinic. Sources included leftover antibiotics from a prior illness (25% vs 9%); from a grocery store (23% vs 5%); or from a family member or friend (17% vs 6%). Knowledge of antibiotic side effects was similar between both groups; however, Hispanics were less aware of antibiotic risks
Gebeyehu E *et al.* 2015^40^ Ethiopia, Northwest Ethiopia, Bahir Dar	1082 Ethiopian households divided into urban areas (*n* = 362) and rural (*n* = 719)	Sex, age, family size, family monthly income, education status including literacy, employment status, marital status, socioeconomic deprivation	Inappropriate use of antibiotics in the community	Proxy: antibiotic use	Quantitative comparative cross-sectional study to determine inappropriate use of antibiotics and its associated factors	Inappropriate antibiotic use was 30.9% with little difference between urban (33.1%) and rural (29.2%) locations. Self-medication accounted for 18% and 12.9% for family member medication. Use of antibiotics included respiratory infections 74.6%, diarrhoea 74.4% and physical injury/wounds 64.3%. Factors associated with inappropriate use included: younger age, low educational status, employment, dissatisfaction with the health services, and antibiotic knowledge
Geta K and Gibret M, 2022^41^ Ethiopia, Amhara Regional State, Northwestern Ethiopia	232 Ethiopian men and women in urban secondary care public hospitals	Age, sex, religion, marital status, level of education status, employment status	Knowledge, attitudes and practice for antibiotics and antibiotic resistance	Proxy: antibiotic knowledge and use	Quantitative cross-sectional survey using a questionnaire. The aim of the study was to assess knowledge, attitudes and practices regarding antibiotic use and resistance	Low level of awareness about antibiotic use and associated resistance. 60.3% of participants purchased antibiotics from a private pharmacy without a prescription. 42.7% stated the reason was shortage of time to be examined by a doctor. 47.9% of patients had taken antibiotics more than five times over 12 months. 69.8% heard about antibiotics and resistance from multiple sources, with 37.5% advised by doctors and nurses. 68.1% lacked knowledge about the causes of transmission of resistant bacteria. However, 10.8% stated the main transmission route was via contaminated water and soil. Multiple linear regression analyses showed education levels were positively correlated with increased levels of knowledge, attitudes and practice on antibiotic use and resistance (*P* < 0.05)
Gunasekera YD *et al.* 2022^42^ Sri Lanka, Western province, Uva	Urban and indigenous rural communities: Urban: female *n* = 132, male *n* = 48 Rural: female *n* = 61, male *n* = 83	Sex, age, education level, urban/rural	Antibiotic understanding and use	Proxy: antibiotic use	Quantitative study including questionnaires and interviews. The aim was to explore the respective communities’ knowledge, attitudes and practices of antibiotics and AMR, including use of antibiotics	Perceptions about participants’ knowledge and actual knowledge (measured via a test question) were correlated (*r* = 0.49, *P* = 0.001) for rural respondents but not for urban respondents. Misconceptions included paracetamol (painkiller), perceived as an antibiotic by more than 50% of both urban and rural respondents. 18.5% of urban and 35.4% of rural participants would keep and reuse what they perceived as leftover antibiotics
Ha TV *et al.* 2019^43^ Vietnam, Kon Tum, Gia Lai, Dak Lak, Dak Nong and Lam Dong	1000 Highland rural households. Kinh dominant ethnicity and other ethnic minorities	Sex, age, marital status, ethnicity, socioeconomic status, education level, occupation	Awareness about resistance, antibiotics and their use	Proxy: antibiotic use	Quantitative household study including a structured questionnaire and face-to-face interviews. The purpose was to explore people’s awareness about antibiotic resistance, antibiotic use and identify associated factors	Higher age, education and family income were positively associated with being aware of prescription medicine, antibiotics and antibiotic resistance. Females had a lower likelihood of being aware of prescription medicine (OR = 0.64; 95% CI = 0.45–0.90) compared with males. Freelancers were more likely to be aware of antibiotic resistance (OR = 2.30; 95% CI = 1.13–4.67) compared with people working in agriculture/fishery/forestry sectors. Most of the ethnic minorities were less likely to be aware of prescription medicine, antibiotics and antibiotic resistance in comparison with the Kinh ethnic group. People who were Mnong ethnic were more likely to know of prescription medicines but less likely to know about antibiotic resistance
Haenssgen MJ *et al.* 2019^16^ Laos; Thailand, Chiang Rai Salavan	2141 men and women living in rural villages	Sex, age, ethnicity, nationality, education level, employment status, religion, cultural practices, e.g. use of traditional healer	Access to health services	Proxy: use of antibiotics	Aims were to (i) describe antibiotic-related knowledge, attitudes and practices; and (ii) assess the role of antibiotic-related knowledge and attitudes in antibiotic access from different providers. Quantitative study using a face-to-face questionnaire	Rural populations showed varying levels of awareness and attitudes, with 87.6% referring to antibiotics as anti-inflammatory drugs. However, some demonstrated high levels of knowledge corresponding to AMR awareness training. Access to antibiotics through informal sources was relatively low. Counterintuitive links were observed between informal antibiotic use, socioeconomic status and attitudes towards AMR. In Salavan there were high rates of informal antibiotic use. The importance of context-specific AMR communication strategies and interventions that target upstream drivers of antimicrobial resistance, antimicrobial use and social economic factors were stated
Halfvarsson J *et al.* 2000^44^ Viet Nam, The Uong Bi District, Quang Ninh Province in the north-east of Vietnam	249 mothers of children under 5 years old and drug vendors from nine different ethnic groups located in rural areas	Socioeconomic conditions, ethnicity, local cultural practices including traditional Vietnamese medicine	Perceptions and use of antibiotics for treatment	Respiratory tract infection	A combination of qualitative and quantitative methods. The aim was to assess mothers’ perceptions and use of antibiotics specifically for the stated infection in children	90% of participants stated antibiotics had negative effects—the child becoming thinner (46%), slowed growth of the child (45%) and caused tiredness (18%). Complications, e.g. diarrhoea, only stated by 6%. Respiratory infection caused by change in temperature not bacteria. Majority of participants treated for 3 days or less with 60% self-medicating. Mothers stated vendors advised limited course, in contrast to the vendors advising 7 day course. Economic conditions influenced purchase. Easy access to antibiotics allowed the purchase of small doses and did not affect the mothers’ work practice
Hernandez-Diaz I *et al.* 2019^45^ Puerto Rico, San Juan East and West Puerto Rico	101 Latino parents of children under 6 years old in an urban secondary care hospital including the emergency department	Ethnicity, level of medical insurance, level of education, age, number of children	Antibiotic use and preparation for educational tool	Upper respiratory tract infection	Cross-sectional study. The aim was to evaluate parents’ or legal guardians’ knowledge, beliefs, behaviours and adherence for antibiotic use	Antibiotics were available without prescription. Poor knowledge and incorrect beliefs were seen in the parents/guardians. There were suboptimal scores in the knowledge and beliefs domain, indicating lack of knowledge of how antibiotics work and what types of infection they treat. Parents/guardians of a younger age with a lower level of education and Medicaid insured were less likely to answer the knowledge questions correctly
Hika K *et al.* 2022^46^ New Zealand, Apakura Marae, Papakura	30 Māori men and women aged 20 to 77	Systemic factors: effect of colonization. Social factors: knowledge, poverty barriers. Individual factors: illness perceptions and treatment beliefs. Health literacy	Systemic factors: GP times and ratios. Social factors: access to healthcare, relationship with health professionals. Individual factors: natural vs Western medicine	Upper respiratory tract infection	A qualitative study. Overall aims were: (i) to examine the experiences, perceptions and beliefs that Māori people have about antibiotics; (ii) to assess their use of antibiotics for acute upper respiratory tract infections; and (iii) to assess knowledge about antimicrobial resistance	Three themes with seven sub-themes outlined under social determinants and health-seeking behaviour acted as the framework for the research. Systemic factors: participants with chronic conditions and additional infections were not always addressed due to lack of GP time/availability. Historical colonization: participants described systems supporting European New Zealanders rather than Māori traditional practices, which exacerbated health literacy. Removal of traditional Rongoa (healing) knowledge impacted healthcare utilization and perceptions of medicine. Urbanization had also impacted their health-seeking behaviour and integration into health and societal systems
Irawati L *et al.* 2019^47^ Malaysia, Jelutong District, Penang	22 urban community residents. Including Malay, Chinese and Indian ethnic groups	Sex, age, ethnicity, marital status, education level, employment status, monthly household income, socioeconomic deprivation	Obtaining antibiotics	Proxy: antibiotic knowledge and perceptions	Qualitative study using semi-structured interviews. The aim was to explore knowledge, attitudes and perceptions about antibiotics and antibiotic resistance. A secondary aim was to identify areas to be addressed when designing an educational intervention to increase residents’ knowledge and change their attitudes and perceptions	Participants had different levels of understanding about antibiotics and antibiotic use. The majority believed antibiotics kill viruses and can speed recovery from viral infections. Most were unaware antibiotics can have adverse effects. The majority reported obtaining antibiotics from a trusted physician and were advised how to take the medication. Some acquired antibiotics from a community pharmacy prior to consultation as they wanted to recover quickly from viral infections. A few took antibiotics given to them by their family or friends, to treat colds and sore throats. More than half adhered to antibiotic regimens. However, treatment was discontinued when symptoms improved, as they were unaware about taking the full course and believed they had recovered
Khan FU *et al.* 2020^48^ Pakistan, Swat, Khyber-Pakhtunkhwa	399 Pakistani men and women attending pharmacies in an urban, post-conflict, region	Socioeconomic deprivation, age, sex, level of education, occupation	Obtaining antibiotics from pharmacies	Proxy: antibiotics	Qualitative, cross-sectional study. The aim was to assess knowledge, attitudes and practices for antibiotics and antibiotic resistance	Most of the participants were male (aged 34 to 41). Some were uneducated (32.1%), and most were unemployed (87%). Most 269 (67.4%) knew the term antibiotic. There was poor knowledge; 334 (83.7%) didn't know the related terminology, and 285 (71.4%) had not heard about antibiotic resistance
Kong LS *et al.* 2019^49^ Malaysia, Kuala Lumpur	402 urban tertiary hospital patients: male (*n* = 201) and female (*n* = 201). Aged 60 years and over. Ethnicities: Malay *n* = 233 (58%); Chinese *n* = 111 (27.6%); Indian *n* = 55 (13.7%); and other *n* = 3 (0.7%)	Age, ethnicity, educational level, occupation and employment status related to healthcare, monthly income	Knowledge, expectations and sources of antibiotics	Proxy: antibiotics.	Aim was to assess participants’ knowledge of antibiotic use and their expectations towards the need for antibiotics and the relationship between an older age and the outlined factors. Quantitative cross-sectional study	70.4% of participants knew antibiotics treated bacterial infections. However, there was a lack of knowledge about AMR and inaccuracies—believing antibiotics treat viral infections 53.5%; and 53.7% believed they treat coughs and colds. Inappropriate use of antibiotics observed, including storage of pre-used antibiotics at home. 266 (66.7%) participants didn't follow the actual prescribed dose regimen. Older people trusted the decision of the doctor more than younger people did
Larson EL *et al.* 2006^50^ USA, Manhattan	30 participants. Group 1 (*n* = 6): urban community members not in formal health system. Group 2 (*n* = 19): participants with some health insurance, plus Latino women with Spanish as primary language, households with one preschool child, and residents of region. Group 3 (*n* = 5): two independent store staff, three healthcare staff	Socioeconomic deprivation, education status, health literacy, costs for healthcare (health insurance Medicaid)	Ease of access to purchase antibiotics from a bodega	Self-prescription of antibiotics for multiple conditions including upper and lower respiratory tract infections and wounds	Mixed methods exploratory descriptive study. Described knowledge, attitudes, beliefs and practices of community members’ use of antibiotics	Predisposing factors: lack of knowledge, cultural attitudes and misconceptions about antibiotics treating other conditions, e.g. asthma, pain and allergies. Health information obtained from friends and family. Factors reinforced ease of self-prescribing antibiotics. Enabling factors: included socioeconomic and ease of access to cheap antibiotics through bodegas and other independent stores were identified as a tradition of care. Despite it being illegal to sell antibiotics, bodega employees and pharmacists were seen as part of the community. People were not prepared to wait in conventional medical settings. Reinforcing factors: when symptom relief occurred, self-prescribing was reinforced and repeated for different illnesses
Lindenmeyer A *et al.* 2016^51^ UK, national	*n* = 23 migrants with Iranian, Polish, Pakistani, Indian, Iraqi, African, Chinese ethnicity who had been in the UK for >1 year, but <5 years	Ethnicity, age, immigration status, reason for move and language	Seeking antibiotics	Proxy: antibiotic use	Qualitative interviews were undertaken with the aim to obtain maximum variation in the sociodemographic factors	Antibiotics were available over the counter and on prescription. Some participants reported people on a low income using unregulated sellers. Key themes included: the curative power of antibiotics, ‘strong’ medicines and getting better quickly if administered IV. Belief in the importance of obtaining antibiotics quickly caused frustration because of delays in obtaining antibiotics via the UK health system. Frustration and concern about being misunderstood by the GP was caused by ‘watch and wait’ and prescriptions of paracetamol rather than antibiotics. Unsatisfactory experience of accessing primary care influenced seeking assistance from friends and relatives from home or accessing migrant networks in the UK
Mason T *et al.* 2018^52^ UK, Greater London	Stage 1: the public from affluent areas (*n* = 384); stage 2: the public from deprived areas (*n* = 384). Community pharmacists (CPs) from both areas (*n* = 240). Age range 18 to 65. Ethnicity classified as White and non-White. Urban area	Ethnicity, language, education, profession, profession of family member ± healthcare profession, campaign exposure, socioeconomic deprivation	Obtaining antibiotics and knowledge of AMR	Proxy: obtaining antibiotics	Quantitative cross-sectional study. The aim was to assess the awareness and knowledge of antibiotic usage and antibiotic resistance	Response rate: affluent areas *n* = 139/384 (36%); deprived areas *n* = 220/384 (57%); CPs *n* = 60/240 (25%). Knowledge of how antibiotics work could not be distinguished between affluent and deprived areas. However, residents in affluent areas had better understanding of antibiotic resistance and were prudent in the use of antibiotics—both statistically significant. Following awareness campaigns knowledge of antibiotic resistance was not increased, and only partial increase in knowledge of antibiotic usage. In deprived areas only 20% had received input from a CP; however, 74% had taken antibiotics on at least one previous occasion. Adherence was improved following counselling by a CP. However, no improvement following awareness campaigns
McNulty C *et al.* 2019^53^ UK, England	2283 adults over 15 years old including 777 parents with children under 5 years old. General public community venue across both rural and urban areas	Age, sex, social grade, education level, children under 15 years old in household, attendance at GP/pharmacy in previous 12 mo, ethnic grouping. Health literacy	Antibiotic use	Antibiotics used for: respiratory tract infection including upper and lower respiratory tract infections, urinary tract infection and cystitis	Quantitative cross-sectional study. The aim was to understand public understanding and use of antibiotics	Understanding had not changed substantially over the past 14 years. Questions on antibiotic resistance showed uncertainty. Respondents trusted GPs more than nurses/pharmacists. Higher social grade and qualifications were strongly positively associated with knowledge of antibiotics and antimicrobial resistance. Households with children or who visited a doctor/pharmacy in the preceding 12 mo were more knowledgeable; youngest (15 to 24 years), oldest (65 + years) and Black, Asian and minority ethnic adults were less knowledgeable. 43% (568/1319) did not receive advice when they had antibiotics. Most people had a basic understanding, misunderstandings continued about knowledge and keeping antibiotics ‘just in case’
McNulty C *et al.* 2022^54^ UK, England	2022 adults aged 15+ including 521 Black, Asian and minority ethnic (BAME) participants and *n* = 406 aged 15 to 25years	Age, sex, social grade, level of education/reached, qualification, ethnic grouping	Antibiotic use	Proxy: antibiotics	Quantitative, cross-sectional study to describe public attitudes and knowledge around antibiotic activity, resistance and use	84% of participants would be pleased if their GP informed them they did not require antibiotics. Trust in GP’s decision about prescribing antibiotics remains high (89%); trust in nurses (76%) and pharmacists (71%). 21% would challenge an antibiotic decision; this was higher for BAME participants (OR 2.5; 95% CI 1.89 to 3.35). 70% had received advice for antibiotics. Belief in benefits of using antibiotics for ear infections was high (68%). Social grade DE and BAME participants, and people with less education had significantly less understanding about antibiotics and resistance
Norris P *et al.* 2009^55^ New Zealand, Auckland and Wellington	Samoan men and women Stage 1: 13 interviews conducted. Stage 2: 112 people attending the healthcare facilities in urban areas completed the questionnaire	Ethnicity, age, sex, culture	Use of antibiotics	Antibiotic use for multiple conditions including upper and lower respiratory tract infections	Qualitative in-depth interviews. The aim was to investigate understandings and use	Many participants had little understanding of antibiotics. Less than 2% identified the correct purpose, 66% thought they were used to relieve pain. Antibiotics frequently confused with paracetamol, analgesics and other medication. Confusion was also evident in respondents who had correctly stated antibiotics were medicines to kill bacteria. Some people received advice from health professionals that sometimes appeared to have changed attitudes or behaviour. Some perceived doctors had become less likely to prescribe antibiotics. Participants reported stopping taking antibiotics before finishing the course. Very few (8%) were aware of antibiotic resistance. Healthcare practitioners cannot assume everyone has a Western view about antibiotics as Samoans may have traditional views
Paredes JL *et al.* 2022^56^ South America, Peru	231 Peruvian parents based in rural areas including jungle and highland primary health centres	Sex, age, socioeconomic deprivation, education status, number of children, age of the older child	Obtaining antibiotics including without prescription	Proxy: antibiotic use behaviour	Cross-sectional study across six centres. The aim was to describe the knowledge, attitudes and practices	183 parents (79%) did not know that antibiotics cannot cure viral infections. 185 participants (80%) did not disagree with wanting their child to receive antibiotics and would not be satisfied if the doctor refused to prescribe antibiotics. Half of parents (*n* = 120, 52%) reported self-medicating their child with antibiotics. Parents more likely to have purchased antibiotics without prescription and to have received antibiotics after recommendation from a pharmacist. A positive correlation was found between knowledge and attitudes after adjusting for the age and the education of the parent. Parents <20 years old were more likely to have low knowledge about antibiotics compared with those aged >40 years
Pattnaik M *et al.* 2023^57^ India, Odisha, Tigiria	1003 households of adult men and women aged from 25 years. Rural villages	Age, sex, education level, occupation, family type including joint, single, nuclear and extended. Ethnicity including general, schedule caste, schedule tribe, other backward castes	Healthcare utilization and antibiotic use	Proxy: antibiotic use	Quantitative, cross-sectional study to assess knowledge, attitudes and practices about antibiotic behaviour and antimicrobial resistance	Most participants aged 18 to 45. Higher proportion of female participants. 208 (20.7%) participants had no formal education, and 59% belonged to backward castes. 446 (44.47%) study participants had heard about antimicrobial medicines and only 0.10% of study participants knew about antimicrobial resistance. 28.03% took antibiotics for colds, 14.75% purchased antibiotics over the counter without prescription, and 20.14% of participants stopped taking antibiotics before completing the full course
Russom M *et al.* 2021^58^ Eritrea, Gash Barka, Debub, Keih-Bahri, Anseba, Debubawi Keih-Bahri and Maekel	2477 men and women aged over 18 years living in 13 urban places	Age, sex, religion, residence location (zone), education level, household size, occupation	Use and knowledge of antibiotics	Proxy: antibiotics	Quantitative cross-sectional study to measure knowledge, attitudes and practices of antibiotics and identify key determinants. Data were analysed in Statistical Package for the Social Sciences (SPSS).	23.8% of participants had taken antibiotics without prescription and/or discontinued the course. Self-medication was linked to non-serious disease, quick relief of symptoms, successful previous experience, shortage of time to visit health facilities and long queues for treatment. Age 24 years or less, male sex, higher education level and poor attitude score were determinants of inappropriate practice. Information was from health facilities (39.6%), television (31.5%) and other people (10.1%). 78.2% reported antibiotic resistance could affect them and their family, and 63.6% correctly answered the definition of antibiotic resistance. Knowledge was higher in males, Christians, people aged between 25 and 54 years, higher educational level, large family size and government employees. 1473 had animals; 14.0% had treated their animals with antibiotics at least once
Schuts EC *et al.* 2019^59^ Netherlands, Amsterdam	21 617 men and women from the Healthy Life in an Urban Setting (HELIUS) study. Six ethnic groups including: Dutch, Surinamese, Ghanaian, Moroccan, and Turkish. Urban areas	Sex, age, ethnicity, migration generation, education level, marital status, health status including 13 health conditions, smoking and alcohol history, Dutch language ability, perceived health	Behaviours associated with antibiotic use	Proxy: understanding of antibiotic use during influenza-like illness, pneumonia, fever, sore throat and bronchitis	Quantitative cross-sectional study. Aim to determine whether appropriate knowledge and use of antibiotics differ by ethnicity and whether knowledge of antibiotics is associated with antibiotic use	All ethnic minority groups had a lower level of antibiotic knowledge compared with the Dutch population. However, the effect for second-generation Ghanaians was not statistically significant. Antibiotic knowledge was higher in all age groups >25 years of age (except for over 65s) compared with under-25s. Women had significantly higher odds of having a higher level of antibiotic knowledge compared with males. Lower knowledge was associated with certain medical conditions, individuals who regularly or occasionally requested antibiotics or did not finish treatment. However, a lower level of antibiotic knowledge was not associated with receiving antibiotics or number of antibiotic prescriptions. Ethnic differences in antibiotic use could not be explained by level of antibiotic knowledge
Sindato C *et al.* 2020^60^ Tanzania, Ilala, Kilosa and Kibaha districts	828 men and women from urban and rural households. Participants across three regions	Sex, age, marital status, education level, employment status, income source	Knowledge and behaviours associated with antibiotic use	Proxy: antibiotics	Quantitative cross-sectional community-based study to determine the knowledge, attitudes and practices regarding antibiotic use and AMR among people with different livelihoods	There was a high level of understanding about antimicrobials but only a moderate level of KAP for antimicrobial use (AMU) and AMR. Age and level of education were significantly linked with the person’s KAP score. The majority (95.3%) had no formal education and had lower levels of awareness than people with other levels of education. Combined AMU knowledge and attitude scores had a better influence on AMU practice scores than independently. People aged 48 to 95 years old reported antimicrobial choice was influenced by health providers. However, younger people, people not married or separated were less likely to consult health workers. Self-medication was evident in a range of conditions and was 24.4% for UTI
Ulaya G *et al.* 2022^61^ Vietnam, Ha Nam Province, Northern region	324 men and women from rural households	Age, sex, education level, occupation, household wealth tertile, usual health facility, distance to health facility, medical insurance, frequency of media use, source of health information	Health-seeking information	Proxy: antibiotic use	Quantitative, cross-sectional study to assess the levels of awareness and knowledge of antibiotics and antibiotic resistance. Investigated the determinants of awareness and knowledge to inform the development of interventions	71.8% of households were aware of antibiotics; however, recognition of antibiotics was much lower 21.6%. Awareness about antibiotic resistance was also low at 18.2%. People using private clinics, pharmacies, or drugstores as their usual healthcare provider were more familiar with antibiotics than people who used government facilities, but less aware of antibiotic resistance. Living more than 10 min away from health facilities was linked with lower antibiotic awareness. Television, consulting health workers and community radio were most frequently used for health information. There was an association between media use and increased awareness of antibiotics and antibiotic resistance
Wang Y *et al.* 2022^62^ China, Eastern China: Zhejiang and Jiangsu provinces	1494 (*n* = 1379 eligible) men and women in two rural villages	Sex, age, marital status, education years, occupation, annual household income, chronic disease history	Obtaining antibiotics	Proxy: antibiotics	Quantitative, cross-sectional study aimed to understand antibiotic use and access patterns and their influencing factors	667 (48.4%) of the 1379 eligible participants had taken antibiotics over a year and this varied with marital status and age group. 59.9% of participants obtained antibiotics from medical facilities with a prescription. Other routes included pharmacies and online purchasing. People who obtained antibiotics not via a conventional route included people aged 15 to 44, unmarried, non–white collar workers with higher years of education, lower annual household income and lower levels of antibiotic knowledge
Westerling R *et al*. 2020^63^ Germany, Netherlands, Turkey, Sweden	130 men and women in Turkey and Turkish migrants in the respective countries. Family physicians and pharmacists	Citizens: sex, age, educational level. Family physicians: sex, age, professional experience. Pharmacists: sex, age, professional experience	Antibiotic use and policy review of health systems across several countries	Proxy: antibiotic use	Qualitative data were collected with the aims to explore the variation in implemented policies and the perceived access to antibiotics, and information on antibiotic use among migrants living in the three European countries	Knowledge of correct antibiotic use was lower and requests for antibiotics were higher among lower socioeconomic groups. However, participants were interested in learning about antibiotics from physicians and via different media routes. Access to antibiotics included without a prescription, brought from host country, online and leftover tablets from family/others. Friends/families were seen as important, and mothers managed medications within families. According to participants, physicians did not provide adequate information. However, physicians stated they provided mainly verbal information, but patients did not understand or want to use medicine when feeling better
Whittaker A *et al.* 2019^64^ Australia, Melbourne	31 ethnic inpatients and interpreters in an urban public hospital and ethnic community members. Ethnicities included Chinese, Afghan, Chilean, Ethiopian, Samoan, South African, Indian, French, German, Pakistani, Pacific Islands, Sri Lankan, Thai, Dutch, New Zealand Vietnamese and Sudanese	Age, sex, country of origin	Hospital treatment, seeking treatment abroad, traditional remedies	Upper respiratory tract infection	The aim was to outline the understandings and experiences of people with antibiotic use and AMR to inform public antimicrobial stewardship and education programmes. Qualitative, semi-structured face-to-face in-depth interviews	Overall poor understanding of AMR with causes attributed to weather and climate change, poor environmental cleanliness and transfer by migrants. Asian and Middle Eastern people referred to the humoral effect, with antibiotics causing imbalance. Antibiotics seen as being ‘Western’ and strong, which should be used sparingly, and preference given to home remedies. Other themes included climatic changes increasing bacteria, increased mobility, new migrants ‘spreading bugs’, hygiene, local prescribing culture, resistant bodies and lack of literacy
Xu Y *et al.* 2020^65^ China, Zhejiang and Shaanxi	2924 parents from Zhejiang and 3355 parents from Shaanxi whose children were 0 to 13 years old living in both rural and urban areas	Sex of the child, age of the child, sex of the parent, parents’ level of education, parents’ medical background, monthly household income, residential location	Use of antibiotics	Proxy: antibiotics	Quantitative cross-sectional study to assess antibiotic use in children in one developed and one less developed region to identify regional parental behaviour disparities	Children in less developed regions faced higher risks of antibiotic misuse. After adjusting for sociodemographic factors, parents in Shaanxi were more likely to engage in self-medication for children than those in Zhejiang (OR = 2.82; 95% CI 2.06–3.86). Although there were no significant regional differences for requesting antibiotics, parents attending paediatric consultations in Shaanxi were more likely to receive prescribed antibiotics than in Zhejiang (OR = 1.46; 95% CI 1.16–1.84). Parents in Shaanxi were more likely to keep antibiotics at home for children (OR = 1.93; 95% CI 1.72–2.17) and to administer prophylactic antibiotics to their children (OR = 1.82; 95% CI 1.58–2.10)

### Knowledge

Knowledge of AMR was assessed in 3/40 studies from Australia,^64^ Sri Lanka^42^ and Malaysia.^47^ Sociodemographic factors associated with better knowledge of AMR included living in an urban area and having a higher level of education and occupation.^42,60^ Misconceptions about AMR^64^ have been exacerbated by a lack of information in people’s principal language and the difficulty in translating key words, such as antibiotics and AMR, into comparable terms in local languages. These language challenges have impacted the assimilation of knowledge^64^ and people’s behaviour when purchasing antibiotics.^66^

Knowledge of antibiotic resistance (ABR) was assessed in 20/40 studies. Although some participants knew the term ABR, they were unable to fully discuss the concepts and connect antibiotic misuse with ABR.^31,33,34,41^ Comprehension by participants of the risks of ABR and its impact on human and animal health was poor, as was the role they can play in prevention.^41,47,63^ Greater knowledge was found in people who had obtained information from the media including social media,^61^ or who came from higher socioeconomic backgrounds, were of White ethnicity^53^ and aged between 45 and 64 years.^36^ Although migrant and refugee populations had not previously been aware of ABR, their knowledge increased following interaction with health professionals in host countries.^30,36^ Use of social media for health promotion needs to be carefully monitored to prevent misinformation, as occurred in the Covid-19 pandemic, which affected vaccine uptake and misinformation about the use of repurposed medication.^67^  ^,68^

Antibiotic knowledge was assessed in 30/40 studies and demonstrated overall poor knowledge. There were considerable variances in people’s understanding about antibiotics, their uses and consumption requirements, ranging from no knowledge about the antibiotics they had taken^33^ to having a reasonable understanding.^48,49^ Participants were unable to provide specific examples of bacterial infections and discussed antibiotics as antivirals and anti-inflammatory drugs.^29,30^ Antibiotic recognition tests were included in 10/40 studies and participants had difficulties in correctly identifying the antibiotics, and confused antibiotics with other drugs, such as antidiabetic medication.^55^  ^,58,60^ People identified antibiotics by colour and formulation, e.g. capsules, which caused confusion when antibiotics were the same colour as other medication, e.g. painkillers.^28^ Lower levels of antibiotic knowledge were associated with lower health literacy^11^ and language constraints.^16,38,48^

Information sources included doctors, healthcare staff, and family and friends. Although doctors were seen as trusted sources of information, there was consensus that the information was not easily understood, time was required for explanations, information was given verbally and was not reinforced with hard copy. There was a lack of information in different languages and there were inconsistencies in participants receiving the advice from medical practitioners.^32,42,46,50^ Family and friends, particularly mothers, were considered to be supportive and being responsible for medication when people were ill;^32,33,52,58,63^ however, sometimes incorrect information was given by mothers, e.g. advising antibiotic use for toothache.^50^ Other sources of information included the television, the internet and pamphlets, although use of the media was not associated with increased knowledge in one study.^58,61^

### Attitudes and beliefs

Assessment of attitudes included: (i) acquiring antibiotics and adhering to antibiotic therapy,^47,48,56,60^ (ii) antibiotic access including the individual’s belief in the antibiotics being prescribed,^16,47,56^ (iii) self-medication,^33^ and (iv) AMR.^57,60^ People’s lack of knowledge about antibiotics appeared to affect their attitudes towards the medication. Key themes included: (i) the power of antibiotics, i.e. being seen as a ‘cure all’ and if not taken, incurable disease would develop;^29,30^ and (ii) duration of illness and speed of potential recovery from illness impacted behaviour about using antibiotics for prevention of illness, including fertility issues, not completing antibiotic courses and taking antibiotics to allow attendance at work.^29,30,34,51^ Positive experiences of taking antibiotics, including beliefs about improving and strengthening the body, reinforced the necessity for antibiotics,^34^ and the risks of inappropriate antibiotic consumption and AMR were not perceived in all studies. There were conflicting attitudes about the role of the doctor in prescribing antibiotics; some participants, including migrant, refugee and asylum seeker populations, believed that antibiotics should be prescribed irrespective of the doctor’s advice,^30,34^  ^,51^ whereas other participants were prepared to discuss and accept the decision made by the doctor.^49^  ^,63^ Accessing antibiotics from the pharmacy without prescription was part of the accepted relationship between the pharmacy staff and customers.^30,50^ Factors affecting attitude scores, including gender, educational level, occupation and location,^58^ translated into associations with behaviour and inappropriate antibiotic use.^28^

### Practices and rationale for antibiotic access and use

Studies outlined problems of accessing antibiotics through formal channels for e.g. healthcare providers and facilities. Challenges included obtaining an appointment to see a GP/physician and the costs of the appointment and differences in insurance reimbursement for antibiotics.^46^ Medical practitioners and pharmacists were, in some cases, under pressure to prescribe and dispense antibiotics as they feared loss of income.^34^  ^,36^  ^,63^ Poor healthcare access and/or an unsatisfactory experience drove patients to seek treatment and antibiotics from elsewhere.^51,63^

Different routes of access to antibiotics were described across all income-level countries in 23/40 studies and included: without a prescription through pharmacies,^41,48,49,57^ grocery stores,^39^ drug vendors and quacks,^31,44^ using leftover medication,^29,34,35,39,47,49,50,52,55^ sharing antibiotics with family and friends,^29,33,39,49,50,52^ obtaining medication from abroad^50,52,63,64^ and the internet.^52,62,63^ Obtaining antibiotics without a prescription was attributed to distance and poor access to healthcare facilities,^31,60^ socioeconomic factors including transportation costs, and the associated costs of seeing a doctor plus the cost of medication versus simply obtaining the antibiotics.^31,38,46,56,60^ In cases of severe poverty, incomplete courses of antibiotics were purchased and decisions were made between accessing healthcare or having food.^31,34,44,46,50^ Patients described having a lack of time to see doctors, due to their employment and family commitments, which were linked to their socioeconomic status.^41^ Knowledge of the disease, its severity and trust in the medicine supplier were indicators for patients assessing the suitability of self-medication.^28,47,60^

People including migrants and refugees, who were successfully treated with antibiotics, reported using the same antibiotic for future occurrences of illness without seeking medical care.^30,34^ Dissatisfaction with healthcare services showed an association with using antibiotics inappropriately.^40^ In some countries self-medication is the norm, and for migrant, refugee and asylum seeker populations this practice continued due to difficulties in obtaining medication in host countries.^51^ The effects of historical colonization on Māori society were demonstrated by an imbalance in socioeconomic status, access to healthcare and health information. These factors impacted antibiotic knowledge and use.^46^

### Consumption

Self-medication was linked to adverse practices for antibiotic consumption. The authors used the term ‘inappropriate’ use; however, this is not meant to be derogatory or critical. It is acknowledged that in some countries the classification of inappropriate antibiotic behaviour may be the norm due to a range of determinants. Examples are the impact of socioeconomic factors including not being able to afford a full course of antibiotics, and behavioural practices related to family traditions passed on from one generation to another.^28^ Discontinuing a course of antibiotics was reported in 16/40 studies, with the primary reason being feeling better,^30,35,40,50,58^ often despite advice from the doctor/dispenser.^34^ Other behaviours included forgetting the duration and frequency and missing doses due to being busy at work.^32,33,47^ Occurrences of taking antibiotics for incorrect clinical reasons included colds, sore throats, diarrhoea, infertility, after childbirth, menstruation, nausea, itching, allergies, pain, malaria, sores, hernias and adding penicillin to deodorant for applying to burns, bruises and cuts.^28,29,34,50,57^

Practices for children were split, and in some circumstances child health was prioritized above other family members, due to wanting to avoid complications for their children.^30,32,38,50^ In other studies, the practices for children mirrored the adult self-medication practices. Practices included using leftover medication, with parents reducing the dose for children,^34,56,65^ stopping the medication prematurely or only purchasing an incomplete course,^31,44^ incorrect usage for children suffering with coughs or diarrhoea, and believing that antibiotics could be used as a universal treatment and strengthening the child’s immune system.^38^

Some studies identified traditional medicine being preferred as a therapeutic choice and incorporated with conventional medicine.^46,64^ Participants reported turning to traditional medicine after self-medication had not been successful.^60^

Three studies focused on respiratory tract infections. These included Latino and Māori populations and advocated educational and cultural programmes to address literacy and knowledge deficits.^11,45,46^

### Recommendations provided by the studies

The studies provided several recommendations, which were categorized. Studies supported continuation of awareness programmes with associated behavioural change programmes.^32,35–37,39,42–44,48,49,55,57,60–62^ In addition to continuing with educational programmes, studies also recommended targeted approaches. These included: (i) targeted interventions for people affected by low health literacy,^11^ people from low socioeconomic groups, Black, Asian, Mixed ethnicities (BAME), migrants, refugees, asylum seekers and people who have a past history of taking antibiotics;^30,46,51,54^ (ii) increased advice and information during patient consultations;^52,53^ and (iii) targeted messaging in appropriate languages for the public, dispensers and community organizations.^50,64^ Another recommendation in studies was regulating antibiotic dispensing and standardization of medication.^28,29,33,34,63^ Recognizing that despite awareness programmes, knowledge remains poor, three studies recommended different approaches to manage this.^52,53,59^ These included examining the local environment, the financial interest of drug sellers,^65^ examination of the sociodemographic indicators, and healthcare access to identify at-risk groups.^16,38,40,45^

Based on the collective evidence, a SWOT analysis of existing KAP studies in populations affected by SDoH (Table 2) links the findings with the discussion and future recommendations for KAP studies on AMR.

**Table 2.** Strengths, Weaknesses, Opportunities and Threats (SWOT) of the compiled evidence from existing KAP studies related to vulnerable, deprived and marginalized populations

Strengths	Weaknesses
Rich evidence in the studies as outlined in Table 1 across all income-level countries Evidence of formal and informal routes for obtaining antibiotics Evidence of self-medication and the related causes Evidence of lack of accurate knowledge for antimicrobial resistance (AMR) and antibiotic use Highlights specific contextual factors to be considered, e.g. family dynamics, role of women, costs associated with healthcare including self-pay, health insurance and transportation costs	Many knowledge, attitude and practice (KAP) studies examining similar questions but not providing new recommendations Recommendations focus on continuing awareness programmes, despite evidence highlighting behaviour changes are not sustained The studies do not focus or provide recommendations to address the structural determinants of health

Opportunities	Threats
Address the macro level drivers, e.g. access to health and socioeconomic factors via policy recommendations for all countries New approaches including collection of all indicators to provide clear evidence of the priority social determinants of health (SDoH) Increase the use of co-production, particularly in high-risk areas to collaborate with communities on a long-term basis to assess specific contextual requirements Use of theoretical frameworks including Maslow’s Hierarchy of Needs, the Health Belief Model and Sen’s Capability Approach to highlight the factors that require consideration Countries need to act to prevent and address the increase in obtaining antibiotics via unconventional routes	Increased deprivation and health inequalities will increase poor practice—including obtaining antibiotics via family/friends, the internet, outlet stores and not adhering to dispensing by prescription (where relevant) and taking incomplete courses of medication AMR National Action Plans (NAPs) not prioritizing informal antibiotic practices, self-medication and the structural determinants Countries not able to provide sufficient focus on AMR within the current context of global challenges People retaining previous antibiotic behaviours Implementation of research recommendations need to be acted on

## Discussion

This study aimed to analyse the KAP studies undertaken with vulnerable, marginalized and deprived populations, assess the suitability for identifying gaps in the stated population, and provide future recommendations. The identification of ‘what’ is happening in the field is well demonstrated. Studies conducted across all income-level countries reported concern that people’s lack of knowledge and the practice of self-medication are serious risk factors for the exacerbation of AMR.^39,59,64^ One of the targets of the 2024 UN declaration on AMR is that by 2030, 70% of antibiotics prescribed for humans should be from the WHO Access group.^69^ This target will potentially be at risk if self-medication continues. It is therefore important to look for robust solutions to manage antibiotic use practices across different contexts and populations. Despite the rapidly increasing granularity of the evidence since 2006,^50^ the majority of studies continue to recommend (i) undertaking further KAP studies and (ii) advocating the necessity to continue with public awareness campaigns to change people’s behaviour. If this approach was successful, it is argued, populations would be more aware about antibiotics and the risks of AMR, and the practice of self-medication would be declining; however, evidence does not support this. There are reports of increasing antibiotic use and demand, with global calls to mitigate the threat of AMR.^5,70^

Studies conducted in the UK, Thailand and Laos have demonstrated that awareness programmes have not consistently increased knowledge and are not effective in changing behaviour associated with antibiotic use.^16,52–54^ Health awareness campaigns have also led to adverse consequences, with participants having more knowledge and confidence about antibiotics, resulting in people using leftover medication to treat themselves and commencing selling antibiotics.^16,71–73^

Lack of progress raises concerns about continuing to undertake KAP studies without evaluating the knowledge already accrued. Policy makers and researchers must question what additional value is added to the extant literature through these studies. Without a focus on context and specific population needs, recommendations regarding raising awareness and increasing public knowledge may not lead to sustainable change. There is a gap in acknowledging that structural, environmental and socioeconomic drivers, including proximity and ease of accessing healthcare, are fundamental to addressing obtaining and consumption of antibiotics. People have limited control to change their circumstances, e.g. the lack of access to universal healthcare or access to education.^74^

Despite the sustainable development goals, global deprivation and health inequities have increased.^75–78^ Vulnerable populations affected by low socioeconomic status and inadequate access to WASH are at greater risk of AMR.^79^ Invariably these populations do not have access to universal healthcare and are required to make decisions e.g. between food or medication.^46^ Maslow’s Hierarchy of Needs model includes three categories of need: (i) physiological and safety needs, (ii) love and belonging needs and (iii) self- esteem and self-actualization needs.^24^ This model has been used in healthcare research to examine ways of improving specific patient requirements.^80^ A review of the model, in the context of KAP studies related to vulnerable populations, illustrates that affected populations are trapped at the basic physiological and safety needs level and are unable to aspire to higher levels until they can meet these basic needs. Examination of the Health Belief Model^22^ demonstrates the individual component parts of theory to practice that need to be evident for behaviour change to occur.^81–83^

Amartya Sen’s Capability Approach^25^ offers a powerful alternative framework for understanding antibiotic use and AMR-related behaviours. Rather than focusing solely on *what* behaviours exist, the Capability Approach interrogates *why* individuals make certain health-related choices and *how* these can be meaningfully altered. Central to this approach is the idea that people’s well-being should be assessed not merely by their behaviours or resources, but by their real freedoms, their capabilities, to achieve valued ways of living. These capabilities are shaped by a range of structural and social factors, including race, gender identity, religion, migration status, income and geography.

The application of these frameworks assists in highlighting the implementation challenges of the SWOT analysis as outlined in Table 2. The findings demonstrate that the challenges are global and not only related to LICs and LMICs. Increasing global health inequalities and socioeconomic challenges^8^ reinforce the requirements for global action and country-/region-specific solutions.

Before future KAP studies are conducted, a critical analysis of whether they are required and what new knowledge will be generated needs to be undertaken. Priority must be given to better understanding and responding to structural determinants of health and access to healthcare. We need to recognize that recommendations from HICs to LMICs related to self-medication, and enforcing dispensing regulations, will not be effective because challenges faced by different populations require targeted and contextualized solutions. Critically, there needs to be a focus on community co-production research to deliver solutions for the public, healthcare professionals and drug vendors. Awareness campaigns should be undertaken if they are focused to the specific requirements of different populations, including local language terminology and communication pathways.^84^ The educational activities should be evaluated for suitability and reach.

### Limitations

The research was part of a larger systematic review and this had the advantage that the studies were retrieved and analysed systematically. This provided robust evidence to undertake the SWOT analysis and provide additional recommendations that provide a different perspective on the value of KAP studies in the defined population. This review focused on vulnerable deprived and marginalized populations, and as was outlined in the systematic review,^19^ identifying the at-risk populations and the country-specific terminology was challenging. There is a possibility that defining these groups may have affected the results of the distribution of publications; however, it has highlighted the challenges that different populations face. The selection of the defined population precludes other population groups and, therefore, this study does not consider the complete range of KAP studies that are available in the field. To provide granular evidence, studies that focused on key component sections of KAP studies were included; however, they may not have been formally categorized as KAP studies. Since research studies highlighted the challenges of language and AMR, having considered only English language publications limits the applicability of the findings.

### Conclusion

Studies examining antibiotic use practices, including formal and informal routes for obtaining antibiotics and consumption practices, identified widespread self-medication practices in LICs and LMICs. Causes of self-medication included: (i) lack of healthcare facilities including poor access and lack of transport; (ii) low socioeconomic status with associated costs for both medical consultation and medication; and (iii) inherent practices including sharing antibiotics and obtaining antibiotics from overseas and online. A major shift in research is required, to move from individual behaviours to considering the social and structural determinants of health. Unless we are able to respond to these upstream factors suboptimal self-medication with antibiotics will not be managed with education efforts alone.

## Supplementary Material

dlaf141_Supplementary_Data

## Data Availability

There was no data sharing plan set out at the beginning of this study. Specific requests for the data for valid academic reasons as judged by the first and last authors will be granted, with an appropriate data sharing agreement, and should be sent to the chief investigator (E.C.). For the purpose of open access, the author has applied a CC BY public copyright licence to any Author Accepted Manuscript version arising from this submission.

## Supplementary data

The systematic review search string^19^ Appendix S1 is available as Supplementary data at *JACAMR* Online.
