# Supplementary material for: Social determinants of health and knowledge, attitude and practice studies on antimicrobial resistance—an evidence review and future direction
Source: JAC Antimicrob Resist. 2025 Sep 4;7(5):dlaf141. doi: 10.1093/jacamr/dlaf141 (PMC12409717; doi:10.1093/jacamr/dlaf141)
Supplement: dlaf141_Supplementary_Data [file dlaf141_supplementary_data.docx]

**Supplementary file:- Appendix S1**

**Social determinants of health and knowledge, attitude and practice studies in antimicrobial resistance – an evidence review and future direction.**

**Alison Shutt, Arunima Sehgal Mukherjee, Vrinda Nampoothiri, Alison Holmes, Esmita Charani**

**Appendix Table S1: Systematic review search string**

**Database:**
Ovid MEDLINE(R) and Epub Ahead of Print, In-Process, In-Data-Review & Other Non-Indexed Citations and Daily <1946 to February 15, 2023>

| **#** | **Query** | **Results from 16 Feb 2023** |
| --- | --- | --- |
| 1 | inequalit*.mp. [mp=title, book title, abstract, original title, name of substance word, subject heading word, floating sub-heading word, keyword heading word, organism supplementary concept word, protocol supplementary concept word, rare disease supplementary concept word, unique identifier, synonyms] | 48,081 |
| 2 | socioeconomic factor*.mp. or Socioeconomic Factors/ | 179,102 |
| 3 | social determinant* of health.mp. [mp=title, book title, abstract, original title, name of substance word, subject heading word, floating sub-heading word, keyword heading word, organism supplementary concept word, protocol supplementary concept word, rare disease supplementary concept word, unique identifier, synonyms] | 14,019 |
| 4 | (cult* determinant* or socio cultural determinant* or cultural value*).mp. or exp Culture/ [mp=title, book title, abstract, original title, name of substance word, subject heading word, floating sub-heading word, keyword heading word, organism supplementary concept word, protocol supplementary concept word, rare disease supplementary concept word, unique identifier, synonyms] | 179,002 |
| 5 | ((social* adj1 depriv*) or psychosocial depriv*).mp. [mp=title, book title, abstract, original title, name of substance word, subject heading word, floating sub-heading word, keyword heading word, organism supplementary concept word, protocol supplementary concept word, rare disease supplementary concept word, unique identifier, synonyms] | 4,730 |
| 6 | (Education or school* or tuition* or train* or education* status or (educat* adj2 level)).mp. or exp education/ | 2,005,870 |
| 7 | literacy.mp. or exp Literacy/ or exp Health Literacy/ or exp Information Literacy/ or exp Computer Literacy/ or exp internet literacy/ or exp ehealth literacy/ | 32,720 |
| 8 | health behavio?r.mp. or exp Health Behavior/ | 367,039 |
| 9 | (Level* of income or salary or pay).mp. [mp=title, book title, abstract, original title, name of substance word, subject heading word, floating sub-heading word, keyword heading word, organism supplementary concept word, protocol supplementary concept word, rare disease supplementary concept word, unique identifier, synonyms] | 57,344 |
| 10 | (employment or unemployment or occupation or work* or work* environment).mp. [mp=title, book title, abstract, original title, name of substance word, subject heading word, floating sub-heading word, keyword heading word, organism supplementary concept word, protocol supplementary concept word, rare disease supplementary concept word, unique identifier, synonyms] | 2,140,603 |
| 11 | social behavio?r.mp. or exp Social Behavior/ or exp Interpersonal Relations/ or interpersonal relation*.mp. or personality characteristic*.mp. [mp=title, book title, abstract, original title, name of substance word, subject heading word, floating sub-heading word, keyword heading word, organism supplementary concept word, protocol supplementary concept word, rare disease supplementary concept word, unique identifier, synonyms] | 603,740 |
| 12 | exp social status/ or exp "social aspects and related phenomena"/ or exp social stigma/ or exp social stratification/ or exp social stress/ or (socioeconomic gradient* or socio economic gradient*).mp. or social status.mp. or social stigma.mp. or social stratification.mp. or social stress.mp. [mp=title, book title, abstract, original title, name of substance word, subject heading word, floating sub-heading word, keyword heading word, organism supplementary concept word, protocol supplementary concept word, rare disease supplementary concept word, unique identifier, synonyms] | 27,230 |
| 13 | (disadvantage* adj1 social*).mp. [mp=title, book title, abstract, original title, name of substance word, subject heading word, floating sub-heading word, keyword heading word, organism supplementary concept word, protocol supplementary concept word, rare disease supplementary concept word, unique identifier, synonyms] | 3,023 |
| 14 | (relig* or faith or creed).mp. or exp religion/ or buddhism/ or christianity/ or hinduism/ or islam/ or judaism/ or "religion and medicine"/ or "religion and psychology"/ | 99,293 |
| 15 | belief.mp. or exp Culture/ | 214,045 |
| 16 | exp Poverty Areas/ or poverty.mp. or exp Poverty/ or exp Child Poverty/ or multi* dimensional poverty.mp. | 70,546 |
| 17 | cultural deprivation.mp. or exp Cultural Deprivation/ | 1,198 |
| 18 | exp Health Equity/ or health equit*.mp. or exp Healthcare Disparities/ or equit*.mp. or health in?quit*.mp. | 64,738 |
| 19 | exp Life Expectancy/ or life expectan*.mp. or life span.mp. or exp Longevity/ | 112,512 |
| 20 | ((social* adj1 exclu*) or Social isolation).mp. or exp Social Isolation/ | 31,866 |
| 21 | exp "Quality of Life"/ or Social aspect*.mp. | 264,110 |
| 22 | exp social change/ or exp superstitions/ or exp taboo/ or social change*.mp. or superstition*.mp. or taboo.mp. | 34,731 |
| 23 | exp social support/ or exp community support/ or exp psychosocial support systems/ or exp social isolation/ or exp social marginalization/ or exp social norms/ or exp social vulnerability/ or exp socialization/ or exp sociodemographic factors/ or exp Interpersonal Relations/ or Social network*.mp. or exp Social Environment/ or exp Social Networking/ | 497,475 |
| 24 | exp gender role/ or gender.mp. | 414,200 |
| 25 | exp social values/ or exp social class/ or exp social mobility/ or exp social factors/ or social class.mp. or social values.mp. | 71,228 |
| 26 | deprivation.mp. or exp food deprivation/ or deprivation.mp. or exp water deprivation/ or index of multiple deprivation.mp. | 99,401 |
| 27 | exp Hygiene/ or exp Sanitation/ or Hygiene.mp. or Sanitation.mp. [mp=title, book title, abstract, original title, name of substance word, subject heading word, floating sub-heading word, keyword heading word, organism supplementary concept word, protocol supplementary concept word, rare disease supplementary concept word, unique identifier, synonyms] | 217,320 |
| 28 | exp residence characteristics/ or exp catchment area, health/ or exp home environment/ or exp housing/ or exp neighborhood characteristics/ or housing.mp. | 114,409 |
| 29 | exp Family Characteristics/ or exp Family Health/ or exp Family Conflict/ or exp Family Relations/ or exp Family/ or famil*.mp. | 1,649,433 |
| 30 | exp Transportation/ or public transport.mp. | 78,232 |
| 31 | exp Hierarchy, Social/ or exp power, psychological/ or exp empowerment/ or intergenerational relations/ or exp maternal behavior/ or exp maternal deprivation/ or exp parent-child relations/ or exp parenting/ or exp paternal behavior/ or exp paternal deprivation/ or exp sibling relations/ or exp family separation/ or exp grandparents/ or exp single-parent family/ | 106,653 |
| 32 | exp Environment/ or exp Social Environment/ or Social Environment.mp. | 1,619,648 |
| 33 | stress, psychological/ or financial stress/ or occupational stress/ or stress.mp. | 1,124,241 |
| 34 | (marginali?ed pop* or marginali?ed communit* or marginali?ed people or marginali?ed person or marginali?ed group*).mp. [mp=title, book title, abstract, original title, name of substance word, subject heading word, floating sub-heading word, keyword heading word, organism supplementary concept word, protocol supplementary concept word, rare disease supplementary concept word, unique identifier, synonyms] | 3,902 |
| 35 | (vulnerable pop* or vulnerable person* or vulnerable people or vulnerable communit* or vulnerable group*).mp. or exp african americans/ or exp amish/ or exp arabs/ or exp asian americans/ or exp indigenous peoples/ or exp jews/ or exp roma/ or exp "sexual and gender minorities"/ or exp vulnerable populations/ or exp homebound persons/ or exp homeless persons/ or exp refugees/ [mp=title, book title, abstract, original title, name of substance word, subject heading word, floating sub-heading word, keyword heading word, organism supplementary concept word, protocol supplementary concept word, rare disease supplementary concept word, unique identifier, synonyms] | 153,048 |
| 36 | (disadvant* communit* or disadvant* people or disadvant* person or disadvant* pop* or disadvant* pop*).mp. or exp disadvantaged population/ [mp=title, book title, abstract, original title, name of substance word, subject heading word, floating sub-heading word, keyword heading word, organism supplementary concept word, protocol supplementary concept word, rare disease supplementary concept word, unique identifier, synonyms] | 15,211 |
| 37 | "Transients and Migrants"/ or asylum seek*.mp. or "Emigration and Immigration"/ or (migrant* or immigrant*).mp. | 74,909 |
| 38 | (Gyps* or traveller*).mp. [mp=title, book title, abstract, original title, name of substance word, subject heading word, floating sub-heading word, keyword heading word, organism supplementary concept word, protocol supplementary concept word, rare disease supplementary concept word, unique identifier, synonyms] | 13,082 |
| 39 | exp disabled persons/ or exp amputees/ or exp persons with mental disabilities/ or exp mentally ill persons/ or exp persons with hearing impairments/ or exp visually impaired persons/ or handicap*.mp. or hear* impair*.mp. or hear* loss.mp. or hear* disorder.mp. or deaf.mp. or dumb.mp. or blind*.mp. or visual impair*.mp. [mp=title, book title, abstract, original title, name of substance word, subject heading word, floating sub-heading word, keyword heading word, organism supplementary concept word, protocol supplementary concept word, rare disease supplementary concept word, unique identifier, synonyms] | 600,309 |
| 40 | low income group*.mp. | 1,014 |
| 41 | (Ethnic* or ethnic minorit* or racial minorit* or Asian or black* or ethnic group* or minorit* group* or race* or racial* or people of colo?r or Racial difference*).mp. or exp "Ethnic and Racial Minorities"/ or black.mp. or exp Black person/ or white.mp. or exp Caucasian/ or exp Southeast Asian/ or exp British Asian/ or exp West Asian/ or exp Asian continental ancestry group/ or exp Central Asian/ or exp East Asian/ or exp Asian American/ or exp Asian/ or asian.mp. or exp South Asian/ or mixed race.mp. or Alaska Native.mp. or exp American Indian/ or exp Alaska Native/ or American Indian.mp. or African American.mp. or exp African American/ or Hispanic.mp. or exp Hispanic/ or latino.mp. or Han chinese.mp. or exp Han Chinese/ or people of colo?r.mp. or exp Aborigine/ or Native Hawaiian.mp. or exp Native Hawaiian/ or Pacific Islander.mp. or exp Pacific Islander/ | 1,047,724 |
| 42 | Gender.mp. or exp "Sexual and Gender Minorities"/ or Sex* orientation.mp. or exp Gender Identity/ or exp Transsexualism/ or Gender reassignment.mp. or exp Transgender Persons/ | 422,375 |
| 43 | urban population.mp. or exp Urban Population/ | 66,580 |
| 44 | rural population.mp. or exp Rural Population/ | 72,842 |
| 45 | (protected character* or incl* health group*).mp. | 24 |
| 46 | hard to reach.mp. | 2,691 |
| 47 | (health* seek* or health seek* behav* or seek* behav*).mp. [mp=title, book title, abstract, original title, name of substance word, subject heading word, floating sub-heading word, keyword heading word, organism supplementary concept word, protocol supplementary concept word, rare disease supplementary concept word, unique identifier, synonyms] | 20,480 |
| 48 | (health* access* or health care access* or health* service or access to health*).mp. or exp health insurance/ or exp health care access/ | 241,447 |
| 49 | exp "Patient Acceptance of Health Care"/ or exp Help-Seeking Behavior/ or Help seek*behavio?r.mp. | 172,189 |
| 50 | Treatment* seeking behav*.mp. | 857 |
| 51 | "delivery of health care"/ or exp after-hours care/ or exp culturally competent care/ or exp delegation, professional/ or exp "delivery of health care, integrated"/ or exp health services accessibility/ or delivery of health care.mp. | 253,450 |
| 52 | exp Health Promotion/ or health promotion*.mp. | 107,721 |
| 53 | exp Health Services Accessibility/ or Human right*.mp. | 146,695 |
| 54 | health* provision.mp. | 1,977 |
| 55 | (healthcare staff or med* care or nurs* care or multidisc* team or health* work* or doctor* or Surgeon or physician* or Nurse* or pharmacist* or interdisciplin*).mp. [mp=title, book title, abstract, original title, name of substance word, subject heading word, floating sub-heading word, keyword heading word, organism supplementary concept word, protocol supplementary concept word, rare disease supplementary concept word, unique identifier, synonyms] | 1,404,066 |
| 56 | exp medicine, traditional/ or exp medicine, african traditional/ or exp medicine, arabic/ or exp medicine, ayurvedic/ or exp medicine, east asian traditional/ or exp medicine, persian/ or exp shamanism/ or traditional medicine.mp. | 56,160 |
| 57 | allopathic.mp. | 2,113 |
| 58 | preventative health*.mp. | 774 |
| 59 | Access to Information/ or "Access to Information".mp. | 11,776 |
| 60 | internet access.mp. or Internet Access/ or Patient Education as Topic/ | 90,275 |
| 61 | Health* facilit*.mp. or exp Health Facilities/ | 908,605 |
| 62 | Social Perception/ or Perception*.mp. | 514,986 |
| 63 | exp Rural Health Services/ or rural.mp. or exp Rural Health/ | 198,121 |
| 64 | urban health.mp. or exp Urban Health/ | 23,910 |
| 65 | exp "referral and consultation"/ or exp remote consultation/ or referral*.mp. or consult*.mp. [mp=title, book title, abstract, original title, name of substance word, subject heading word, floating sub-heading word, keyword heading word, organism supplementary concept word, protocol supplementary concept word, rare disease supplementary concept word, unique identifier, synonyms] | 328,804 |
| 66 | exp Health Behavior/ or behavio?r change.mp. or behavio?r intervention*.mp. [mp=title, book title, abstract, original title, name of substance word, subject heading word, floating sub-heading word, keyword heading word, organism supplementary concept word, protocol supplementary concept word, rare disease supplementary concept word, unique identifier, synonyms] | 373,471 |
| 67 | (co* produc* or public engagement or patient engagement).mp. or exp Patient Participation/ [mp=title, book title, abstract, original title, name of substance word, subject heading word, floating sub-heading word, keyword heading word, organism supplementary concept word, protocol supplementary concept word, rare disease supplementary concept word, unique identifier, synonyms] | 162,820 |
| 68 | community participation.mp. or exp Community Participation/ | 50,336 |
| 69 | Health Education/ or Health Knowledge, Attitudes, Practice/ | 179,924 |
| 70 | exp Attitude to Health/ or belief system.mp. or myth.mp. | 474,809 |
| 71 | Cultural Competency/ or exp "Attitude of Health Personnel"/ or exp Culturally Competent Care/ or exp Cultural Characteristics/ or Cultur* practice*.mp. | 194,601 |
| 72 | Anti microbial stewardship.mp. or exp Antimicrobial Stewardship/ | 3,257 |
| 73 | (infection control or infection prevention control).mp. [mp=title, book title, abstract, original title, name of substance word, subject heading word, floating sub-heading word, keyword heading word, organism supplementary concept word, protocol supplementary concept word, rare disease supplementary concept word, unique identifier, synonyms] | 49,068 |
| 74 | antibiotic resistance.mp. or exp Drug Resistance, Microbial/ | 206,633 |
| 75 | ((antimicrob* adj2 resist*) or anti microb* resist* or AMR).mp. | 42,704 |
| 76 | Drug resistance , multiple, bacterial.mp. or exp Drug Resistance, Multiple, Bacterial/ | 25,227 |
| 77 | (antibact* resist* or anti bact* resist* or ABR).mp. [mp=title, book title, abstract, original title, name of substance word, subject heading word, floating sub-heading word, keyword heading word, organism supplementary concept word, protocol supplementary concept word, rare disease supplementary concept word, unique identifier, synonyms] | 7,719 |
| 78 | exp Anti-Bacterial Agents/ or Anti* bacteria* agent*.mp. or anti* infective.mp. or antimicrobial agent*.mp. or antibiotic*.mp. [mp=title, book title, abstract, original title, name of substance word, subject heading word, floating sub-heading word, keyword heading word, organism supplementary concept word, protocol supplementary concept word, rare disease supplementary concept word, unique identifier, synonyms] | 1,071,207 |
| 79 | Streptococ* pneumoniae infec*.mp. or exp Streptococ* Infections/ or streptococ* infec.mp. or exp Streptococcus pyogenes infec*/ or strep* infec*.mp. or exp Pneumococcal Infections/ or Pneumococ* infec*.mp. | 59,266 |
| 80 | (Staphylococ* infec* or Vancomycin-Resistant Staphylococcus aureus infec).mp. or exp Staphylococcus aureus infec*/ or MRSA infec*.mp. or methicillin* resist* staph* aureus infec*.mp. [mp=title, book title, abstract, original title, name of substance word, subject heading word, floating sub-heading word, keyword heading word, organism supplementary concept word, protocol supplementary concept word, rare disease supplementary concept word, unique identifier, synonyms] | 67,361 |
| 81 | exp Gram-Positive Bacterial Infections/ or gram positive infec*.mp. | 468,880 |
| 82 | gram negative infec*.mp. or exp Gram-Negative Bacterial Infections/ | 396,700 |
| 83 | clostridium infec*.mp. or exp Clostridium Infections/ | 32,444 |
| 84 | (carbapenemase-producing enterobacteriaceae infec* or enterobacteriaceae infections).mp. or exp enterobacteriaceae infections/ | 109,675 |
| 85 | Vancomycin resistant enterococ* infec*.mp. | 136 |
| 86 | exp Acinetobacter Infections/ or Acinobacter infec*.mp. | 5,329 |
| 87 | exp Surgical Wound Infection/ or Surg* infec*.mp. | 41,656 |
| 88 | exp Skin Diseases, Bacterial/ or skin infection.mp. or exp Skin Diseases, Infectious/ | 128,093 |
| 89 | soft tissue infection.mp. or exp Soft Tissue Infections/ | 6,741 |
| 90 | exp Sepsis/ or sepsis.mp. | 207,151 |
| 91 | exp Bacteremia/ or bacter?emia.mp. | 52,153 |
| 92 | exp Urinary Tract Infections/ or urinary tract infect*.mp. | 73,349 |
| 93 | Nosocomial infec*.mp. | 17,287 |
| 94 | (hospital acquired infec* or hospital associated infec* or community acquired infec*).mp. [mp=title, book title, abstract, original title, name of substance word, subject heading word, floating sub-heading word, keyword heading word, organism supplementary concept word, protocol supplementary concept word, rare disease supplementary concept word, unique identifier, synonyms] | 22,703 |
| 95 | exp Respiratory Tract Infections/ or Resp* infec*.mp. or chest infec*.mp. or pul* infec*.mp. or resp* tract infec*.mp. or pneumonia.mp. [mp=title, book title, abstract, original title, name of substance word, subject heading word, floating sub-heading word, keyword heading word, organism supplementary concept word, protocol supplementary concept word, rare disease supplementary concept word, unique identifier, synonyms] | 698,484 |
| 96 | klebsiella.mp. or exp Klebsiella Infections/ | 46,537 |
| 97 | Pseudomonas infec*.mp. or Pseudomonas Infections/ | 22,689 |
| 98 | E coli infec*.mp. or exp Escherichia coli Infections/ | 36,049 |
| 99 | 1 or 2 or 3 or 4 or 5 or 6 or 7 or 8 or 9 or 10 or 11 or 12 or 13 or 14 or 15 or 16 or 17 or 18 or 19 or 20 or 21 or 22 or 23 or 24 or 25 or 26 or 27 or 28 or 29 or 30 or 31 or 32 or 33 | 8,656,901 |
| 100 | 34 or 35 or 36 or 37 or 38 or 39 or 40 or 41 or 42 or 43 or 44 or 45 or 46 | 2,183,961 |
| 101 | 47 or 48 or 49 or 50 or 51 or 52 or 53 or 54 or 55 or 56 or 57 or 58 or 59 or 60 or 61 or 62 or 63 or 64 or 65 or 66 or 67 or 68 or 69 or 70 or 71 | 3,866,334 |
| 102 | 74 or 75 or 76 or 77 | 233,361 |
| 103 | 72 or 73 or 78 or 79 or 80 or 81 or 82 or 83 or 84 or 85 or 86 or 87 or 88 or 89 or 90 or 91 or 92 or 93 or 94 or 95 or 96 or 97 or 98 | 2,498,006 |
| 104 | 102 and 103 | 185,457 |
| 105 | 99 and 100 and 101 and 104 | 740 |
| 106 | exp animals/ not humans.sh. | 5,093,590 |
| 107 | 105 not 106 | 732 |
| 108 | limit 107 to yr="2000 - 2022" | 648 |

inequalit*.mp. [mp=title, book title, abstract, original title, name of substance word, subject heading word, floating sub-heading word, keyword heading word, organism supplementary concept word, protocol supplementary concept word, rare disease supplementary concept word, unique identifier, synonyms]
socioeconomic factor*.mp. or Socioeconomic Factors/
social determinant* of health.mp. [mp=title, book title, abstract, original title, name of substance word, subject heading word, floating sub-heading word, keyword heading word, organism supplementary concept word, protocol supplementary concept word, rare disease supplementary concept word, unique identifier, synonyms]
(cult* determinant* or socio cultural determinant* or cultural value*).mp. or exp Culture/ [mp=title, book title, abstract, original title, name of substance word, subject heading word, floating sub-heading word, keyword heading word, organism supplementary concept word, protocol supplementary concept word, rare disease supplementary concept word, unique identifier, synonyms]
((social* adj1 depriv*) or psychosocial depriv*).mp. [mp=title, book title, abstract, original title, name of substance word, subject heading word, floating sub-heading word, keyword heading word, organism supplementary concept word, protocol supplementary concept word, rare disease supplementary concept word, unique identifier, synonyms]
(Education or school* or tuition* or train* or education* status or (educat* adj2 level)).mp. or exp education/
literacy.mp. or exp Literacy/ or exp Health Literacy/ or exp Information Literacy/ or exp Computer Literacy/ or exp internet literacy/ or exp ehealth literacy/
health behavio?r.mp. or exp Health Behavior/
(Level* of income or salary or pay).mp. [mp=title, book title, abstract, original title, name of substance word, subject heading word, floating sub-heading word, keyword heading word, organism supplementary concept word, protocol supplementary concept word, rare disease supplementary concept word, unique identifier, synonyms]
(employment or unemployment or occupation or work* or work* environment).mp. [mp=title, book title, abstract, original title, name of substance word, subject heading word, floating sub-heading word, keyword heading word, organism supplementary concept word, protocol supplementary concept word, rare disease supplementary concept word, unique identifier, synonyms]
social behavio?r.mp. or exp Social Behavior/ or exp Interpersonal Relations/ or interpersonal relation*.mp. or personality characteristic*.mp. [mp=title, book title, abstract, original title, name of substance word, subject heading word, floating sub-heading word, keyword heading word, organism supplementary concept word, protocol supplementary concept word, rare disease supplementary concept word, unique identifier, synonyms]
exp social status/ or exp "social aspects and related phenomena"/ or exp social stigma/ or exp social stratification/ or exp social stress/ or (socioeconomic gradient* or socio economic gradient*).mp. or social status.mp. or social stigma.mp. or social stratification.mp. or social stress.mp. [mp=title, book title, abstract, original title, name of substance word, subject heading word, floating sub-heading word, keyword heading word, organism supplementary concept word, protocol supplementary concept word, rare disease supplementary concept word, unique identifier, synonyms]
(disadvantage* adj1 social*).mp. [mp=title, book title, abstract, original title, name of substance word, subject heading word, floating sub-heading word, keyword heading word, organism supplementary concept word, protocol supplementary concept word, rare disease supplementary concept word, unique identifier, synonyms]
(relig* or faith or creed).mp. or exp religion/ or buddhism/ or christianity/ or hinduism/ or islam/ or judaism/ or "religion and medicine"/ or "religion and psychology"/
belief.mp. or exp Culture/
exp Poverty Areas/ or poverty.mp. or exp Poverty/ or exp Child Poverty/ or multi* dimensional poverty.mp.
cultural deprivation.mp. or exp Cultural Deprivation/
exp Health Equity/ or health equit*.mp. or exp Healthcare Disparities/ or equit*.mp. or health in?quit*.mp.
exp Life Expectancy/ or life expectan*.mp. or life span.mp. or exp Longevity/
((social* adj1 exclu*) or Social isolation).mp. or exp Social Isolation/
exp "Quality of Life"/ or Social aspect*.mp.
exp social change/ or exp superstitions/ or exp taboo/ or social change*.mp. or superstition*.mp. or taboo.mp.
exp social support/ or exp community support/ or exp psychosocial support systems/ or exp social isolation/ or exp social marginalization/ or exp social norms/ or exp social vulnerability/ or exp socialization/ or exp sociodemographic factors/ or exp Interpersonal Relations/ or Social network*.mp. or exp Social Environment/ or exp Social Networking/
exp gender role/ or gender.mp.
exp social values/ or exp social class/ or exp social mobility/ or exp social factors/ or social class.mp. or social values.mp.
deprivation.mp. or exp food deprivation/ or deprivation.mp. or exp water deprivation/ or index of multiple deprivation.mp.
exp Hygiene/ or exp Sanitation/ or Hygiene.mp. or Sanitation.mp. [mp=title, book title, abstract, original title, name of substance word, subject heading word, floating sub-heading word, keyword heading word, organism supplementary concept word, protocol supplementary concept word, rare disease supplementary concept word, unique identifier, synonyms]
exp residence characteristics/ or exp catchment area, health/ or exp home environment/ or exp housing/ or exp neighborhood characteristics/ or housing.mp.
exp Family Characteristics/ or exp Family Health/ or exp Family Conflict/ or exp Family Relations/ or exp Family/ or famil*.mp.
exp Transportation/ or public transport.mp.
exp Hierarchy, Social/ or exp power, psychological/ or exp empowerment/ or intergenerational relations/ or exp maternal behavior/ or exp maternal deprivation/ or exp parent-child relations/ or exp parenting/ or exp paternal behavior/ or exp paternal deprivation/ or exp sibling relations/ or exp family separation/ or exp grandparents/ or exp single-parent family/
exp Environment/ or exp Social Environment/ or Social Environment.mp.
stress, psychological/ or financial stress/ or occupational stress/ or stress.mp.
(marginali?ed pop* or marginali?ed communit* or marginali?ed people or marginali?ed person or marginali?ed group*).mp. [mp=title, book title, abstract, original title, name of substance word, subject heading word, floating sub-heading word, keyword heading word, organism supplementary concept word, protocol supplementary concept word, rare disease supplementary concept word, unique identifier, synonyms]
(vulnerable pop* or vulnerable person* or vulnerable people or vulnerable communit* or vulnerable group*).mp. or exp african americans/ or exp amish/ or exp arabs/ or exp asian americans/ or exp indigenous peoples/ or exp jews/ or exp roma/ or exp "sexual and gender minorities"/ or exp vulnerable populations/ or exp homebound persons/ or exp homeless persons/ or exp refugees/ [mp=title, book title, abstract, original title, name of substance word, subject heading word, floating sub-heading word, keyword heading word, organism supplementary concept word, protocol supplementary concept word, rare disease supplementary concept word, unique identifier, synonyms]
(disadvant* communit* or disadvant* people or disadvant* person or disadvant* pop* or disadvant* pop*).mp. or exp disadvantaged population/ [mp=title, book title, abstract, original title, name of substance word, subject heading word, floating sub-heading word, keyword heading word, organism supplementary concept word, protocol supplementary concept word, rare disease supplementary concept word, unique identifier, synonyms]
"Transients and Migrants"/ or asylum seek*.mp. or "Emigration and Immigration"/ or (migrant* or immigrant*).mp.
(Gyps* or traveller*).mp. [mp=title, book title, abstract, original title, name of substance word, subject heading word, floating sub-heading word, keyword heading word, organism supplementary concept word, protocol supplementary concept word, rare disease supplementary concept word, unique identifier, synonyms]
exp disabled persons/ or exp amputees/ or exp persons with mental disabilities/ or exp mentally ill persons/ or exp persons with hearing impairments/ or exp visually impaired persons/ or handicap*.mp. or hear* impair*.mp. or hear* loss.mp. or hear* disorder.mp. or deaf.mp. or dumb.mp. or blind*.mp. or visual impair*.mp. [mp=title, book title, abstract, original title, name of substance word, subject heading word, floating sub-heading word, keyword heading word, organism supplementary concept word, protocol supplementary concept word, rare disease supplementary concept word, unique identifier, synonyms]
low income group*.mp.
(Ethnic* or ethnic minorit* or racial minorit* or Asian or black* or ethnic group* or minorit* group* or race* or racial* or people of colo?r or Racial difference*).mp. or exp "Ethnic and Racial Minorities"/ or black.mp. or exp Black person/ or white.mp. or exp Caucasian/ or exp Southeast Asian/ or exp British Asian/ or exp West Asian/ or exp Asian continental ancestry group/ or exp Central Asian/ or exp East Asian/ or exp Asian American/ or exp Asian/ or asian.mp. or exp South Asian/ or mixed race.mp. or Alaska Native.mp. or exp American Indian/ or exp Alaska Native/ or American Indian.mp. or African American.mp. or exp African American/ or Hispanic.mp. or exp Hispanic/ or latino.mp. or Han chinese.mp. or exp Han Chinese/ or people of colo?r.mp. or exp Aborigine/ or Native Hawaiian.mp. or exp Native Hawaiian/ or Pacific Islander.mp. or exp Pacific Islander/
Gender.mp. or exp "Sexual and Gender Minorities"/ or Sex* orientation.mp. or exp Gender Identity/ or exp Transsexualism/ or Gender reassignment.mp. or exp Transgender Persons/
urban population.mp. or exp Urban Population/
rural population.mp. or exp Rural Population/
(protected character* or incl* health group*).mp.
hard to reach.mp.
(health* seek* or health seek* behav* or seek* behav*).mp. [mp=title, book title, abstract, original title, name of substance word, subject heading word, floating sub-heading word, keyword heading word, organism supplementary concept word, protocol supplementary concept word, rare disease supplementary concept word, unique identifier, synonyms]
(health* access* or health care access* or health* service or access to health*).mp. or exp health insurance/ or exp health care access/
exp "Patient Acceptance of Health Care"/ or exp Help-Seeking Behavior/ or Help seek*behavio?r.mp.
Treatment* seeking behav*.mp.
"delivery of health care"/ or exp after-hours care/ or exp culturally competent care/ or exp delegation, professional/ or exp "delivery of health care, integrated"/ or exp health services accessibility/ or delivery of health care.mp.
exp Health Promotion/ or health promotion*.mp.
exp Health Services Accessibility/ or Human right*.mp.
health* provision.mp.
(healthcare staff or med* care or nurs* care or multidisc* team or health* work* or doctor* or Surgeon or physician* or Nurse* or pharmacist* or interdisciplin*).mp. [mp=title, book title, abstract, original title, name of substance word, subject heading word, floating sub-heading word, keyword heading word, organism supplementary concept word, protocol supplementary concept word, rare disease supplementary concept word, unique identifier, synonyms]
exp medicine, traditional/ or exp medicine, african traditional/ or exp medicine, arabic/ or exp medicine, ayurvedic/ or exp medicine, east asian traditional/ or exp medicine, persian/ or exp shamanism/ or traditional medicine.mp.
allopathic.mp.
preventative health*.mp.
Access to Information/ or "Access to Information".mp.
internet access.mp. or Internet Access/ or Patient Education as Topic/
Health* facilit*.mp. or exp Health Facilities/
Social Perception/ or Perception*.mp.
exp Rural Health Services/ or rural.mp. or exp Rural Health/
urban health.mp. or exp Urban Health/
exp "referral and consultation"/ or exp remote consultation/ or referral*.mp. or consult*.mp. [mp=title, book title, abstract, original title, name of substance word, subject heading word, floating sub-heading word, keyword heading word, organism supplementary concept word, protocol supplementary concept word, rare disease supplementary concept word, unique identifier, synonyms]
exp Health Behavior/ or behavio?r change.mp. or behavio?r intervention*.mp. [mp=title, book title, abstract, original title, name of substance word, subject heading word, floating sub-heading word, keyword heading word, organism supplementary concept word, protocol supplementary concept word, rare disease supplementary concept word, unique identifier, synonyms]
(co* produc* or public engagement or patient engagement).mp. or exp Patient Participation/ [mp=title, book title, abstract, original title, name of substance word, subject heading word, floating sub-heading word, keyword heading word, organism supplementary concept word, protocol supplementary concept word, rare disease supplementary concept word, unique identifier, synonyms]
community participation.mp. or exp Community Participation/
Health Education/ or Health Knowledge, Attitudes, Practice/
exp Attitude to Health/ or belief system.mp. or myth.mp.
Cultural Competency/ or exp "Attitude of Health Personnel"/ or exp Culturally Competent Care/ or exp Cultural Characteristics/ or Cultur* practice*.mp.
Anti microbial stewardship.mp. or exp Antimicrobial Stewardship/
(infection control or infection prevention control).mp. [mp=title, book title, abstract, original title, name of substance word, subject heading word, floating sub-heading word, keyword heading word, organism supplementary concept word, protocol supplementary concept word, rare disease supplementary concept word, unique identifier, synonyms]
antibiotic resistance.mp. or exp Drug Resistance, Microbial/
((antimicrob* adj2 resist*) or anti microb* resist* or AMR).mp.
Drug resistance , multiple, bacterial.mp. or exp Drug Resistance, Multiple, Bacterial/
(antibact* resist* or anti bact* resist* or ABR).mp. [mp=title, book title, abstract, original title, name of substance word, subject heading word, floating sub-heading word, keyword heading word, organism supplementary concept word, protocol supplementary concept word, rare disease supplementary concept word, unique identifier, synonyms]
exp Anti-Bacterial Agents/ or Anti* bacteria* agent*.mp. or anti* infective.mp. or antimicrobial agent*.mp. or antibiotic*.mp. [mp=title, book title, abstract, original title, name of substance word, subject heading word, floating sub-heading word, keyword heading word, organism supplementary concept word, protocol supplementary concept word, rare disease supplementary concept word, unique identifier, synonyms]
Streptococ* pneumoniae infec*.mp. or exp Streptococ* Infections/ or streptococ* infec.mp. or exp Streptococcus pyogenes infec*/ or strep* infec*.mp. or exp Pneumococcal Infections/ or Pneumococ* infec*.mp.
(Staphylococ* infec* or Vancomycin-Resistant Staphylococcus aureus infec).mp. or exp Staphylococcus aureus infec*/ or MRSA infec*.mp. or methicillin* resist* staph* aureus infec*.mp. [mp=title, book title, abstract, original title, name of substance word, subject heading word, floating sub-heading word, keyword heading word, organism supplementary concept word, protocol supplementary concept word, rare disease supplementary concept word, unique identifier, synonyms]
exp Gram-Positive Bacterial Infections/ or gram positive infec*.mp.
gram negative infec*.mp. or exp Gram-Negative Bacterial Infections/
clostridium infec*.mp. or exp Clostridium Infections/
(carbapenemase-producing enterobacteriaceae infec* or enterobacteriaceae infections).mp. or exp enterobacteriaceae infections/
Vancomycin resistant enterococ* infec*.mp.
exp Acinetobacter Infections/ or Acinobacter infec*.mp.
exp Surgical Wound Infection/ or Surg* infec*.mp.
exp Skin Diseases, Bacterial/ or skin infection.mp. or exp Skin Diseases, Infectious/
soft tissue infection.mp. or exp Soft Tissue Infections/
exp Sepsis/ or sepsis.mp.
exp Bacteremia/ or bacter?emia.mp.
exp Urinary Tract Infections/ or urinary tract infect*.mp.
Nosocomial infec*.mp.
(hospital acquired infec* or hospital associated infec* or community acquired infec*).mp. [mp=title, book title, abstract, original title, name of substance word, subject heading word, floating sub-heading word, keyword heading word, organism supplementary concept word, protocol supplementary concept word, rare disease supplementary concept word, unique identifier, synonyms]
exp Respiratory Tract Infections/ or Resp* infec*.mp. or chest infec*.mp. or pul* infec*.mp. or resp* tract infec*.mp. or pneumonia.mp. [mp=title, book title, abstract, original title, name of substance word, subject heading word, floating sub-heading word, keyword heading word, organism supplementary concept word, protocol supplementary concept word, rare disease supplementary concept word, unique identifier, synonyms]
klebsiella.mp. or exp Klebsiella Infections/
Pseudomonas infec*.mp. or Pseudomonas Infections/
E coli infec*.mp. or exp Escherichia coli Infections/
1 or 2 or 3 or 4 or 5 or 6 or 7 or 8 or 9 or 10 or 11 or 12 or 13 or 14 or 15 or 16 or 17 or 18 or 19 or 20 or 21 or 22 or 23 or 24 or 25 or 26 or 27 or 28 or 29 or 30 or 31 or 32 or 33
34 or 35 or 36 or 37 or 38 or 39 or 40 or 41 or 42 or 43 or 44 or 45 or 46
47 or 48 or 49 or 50 or 51 or 52 or 53 or 54 or 55 or 56 or 57 or 58 or 59 or 60 or 61 or 62 or 63 or 64 or 65 or 66 or 67 or 68 or 69 or 70 or 71
74 or 75 or 76 or 77
72 or 73 or 78 or 79 or 80 or 81 or 82 or 83 or 84 or 85 or 86 or 87 or 88 or 89 or 90 or 91 or 92 or 93 or 94 or 95 or 96 or 97 or 98
102 and 103
99 and 100 and 101 and 104
exp animals/ not humans.sh.
105 not 106
limit 107 to yr="2000 - 2022"


<https://ovidsp.ovid.com/ovidweb.cgi?T=JS&NEWS=N&PAGE=main&SHAREDSEARCHID=72l3aDGfDBapRxt0WANsCBrU8mQuwj4wF1Sbesq18NCkHCWHtzhUdzzGP4bZx5RaX>
